# Unveiling abundance-dependent metabolic phenotypes of microbial communities

**DOI:** 10.1128/msystems.00492-23

**Published:** 2023-09-05

**Authors:** Natalia E. Jiménez, Vicente Acuña, María Paz Cortés, Damien Eveillard, Alejandro Eduardo Maass

**Affiliations:** 1 Center for Mathematical Modeling, University of Chile, Santiago, Chile; 2 Center for Genome Regulation, Millennium Institute, University of Chile, Santiago, Chile; 3 Nantes Université, Ecole Centrale Nantes, CNRS, Nantes, France; 4 Department of Mathematical Engineering, University of Chile, Santiago, Chile; Katholieke Universiteit Leuven, Leuven, Belgium

**Keywords:** constrained-based modeling, microbial communities, metabolic plasticity

## Abstract

Constraint-based modeling has risen as an alternative for characterizing metabolism of communities. Adaptations of flux balance analysis have been proposed to model metabolic interactions, which in most cases consider the maximization of biomass production as their objective. In nature, novel essential functions are not directly related to cell growth force communities to display suboptimal growth rates. These suboptimal states allow a degree of plasticity in their metabolism, thus allowing quick shifts between alternative flux distributions as an initial response to environmental changes. In this work, we introduce the abundance-growth space as a representation of metabolic phenotypes of a community. This space is defined by the composition of a community, represented by its members’ relative abundances, and their growth rate. The analysis of this space allows us to pinpoint how critical reactions respond to shifts of the environment, showing where changes in community plasticity occur. Interestingly, it highlights the relevance of the relative abundance of its members in the lost or gain of plasticity. This method is applied to two simple communities that exchange metabolites. A synthetic community of two mutant *Escherichia coli* strains and an environmental bioleaching community composed by *Acidithiobacillus ferrooxidans* Wenelen and *Sulfobacillus thermosulfidooxidans* Cutipay, where only Cutipay consumes organic matter disposed of by the community.

**IMPORTANCE:**

In nature, organisms live in communities and not as isolated species, and their interactions provide a source of resilience to environmental disturbances. Despite their importance in ecology, human health, and industry, understanding how organisms interact in different environments remains an open question.

In this work, we provide a novel approach that, only using genomic information, studies the metabolic phenotype exhibited by communities, where the exploration of suboptimal growth flux distributions and the composition of a community allows to unveil its capacity to respond to environmental changes, shedding light of the degrees of metabolic plasticity inherent to the community.

## INTRODUCTION

In nature, organisms live in communities rather than as isolated species. These communities emerge from interactions (1) and provide a source of resilience to perturbations such as biofilm development (2, 3), flux relation-feeding (4), or community resistance to environmental disturbances (5). The study of these interactions is imperative in critical areas such as ecology (6), health (7), and the biotechnological industry (8). Understanding how organisms interact in different environments is still an open discussion despite their relevance.

The last decade saw the rise of metabolic modeling for formalizing microbial interactions. The metabolic cross-talking between microorganisms notably justifies the metabolic abstraction for sustaining biogeochemical cycles (9). As a natural following, several approaches have been developed for modeling the metabolism of communities (10) using comprehensive genome-scale descriptions for which each organism is described by its inner biochemical machinery (11 – 14). This genome-scale description allows developing of several computational strategies to identify essential metabolic interactions (e.g., SteadyCom [15], RedCom [13], community flux balance analysis [cFBA] [16], and MICOM [microbial community] [17]). When applied in specific contexts, it results in insightful advances like the determination of essential interactions in anaerobic digestion for biogas production (18), cancer (19), and the gut microbiota (20, 21).

These approaches characterize metabolic flux distributions and community compositions using an adaptation of flux balance analysis (FBA) (22), where an objective function, usually biomass production, is optimized (13, 15 – 17, 23, 24). Maximization of biomass is of great interest for growth rate estimation, and it has shown substantial benefits in the biotechnological context that aims at controlling single strains to improve yields for a product of interest (25, 26). However, in environmental communities, organisms should be prepared to face shifting conditions. Hence they increase their fitness by maximizing their growth rate for a subset of possible conditions (27), hence displaying suboptimal growth rates. Moreover, metabolic models do not always account for additional requirements, such as the synthesis of secondary metabolites essential to sustain communities (28) and survival in adverse environments (5). Suboptimal growth solutions allow for resources to be used in these additional functions, as it was observed experimentally for model species in references 29 and 30.

A challenge for studying and predicting metabolic interactions in community models is to propose methods allowing the exploration of alternative metabolic states that a community could display while preserving the practicality of traditional methods like FBA and flux variability analysis (FVA) (31). An idea that can be adapted to community models was proposed initially in reference 32, where metabolic phenotypes of an organism are studied by grouping couples of nutrient fluxes by their incidence in the optimal solution of an FBA for biomass. More recently, an alternative approach is proposed to directly explore the flux space defined by exchange reactions to characterize the so-called metabolic niche (33). Comparison between this space for different organisms allows us to determine environments where a community can thrive. However, these approaches still leave out the role and abundance of each organism in the community. Indeed, the relative abundance of members of a community shape the distribution of resources and synthesis of secondary metabolites (15). Although there are promising ideas regarding the laws governing its probabilistic distribution (34), the prediction of abundances is uncertain at steady state. Thus, the exploration of the effect of community composition is critical for studying metabolic interactions.

In this work, we explore the space of metabolic fluxes of a community, focusing on the distribution of abundances of its organisms and community growth rates. This space is partitioned according to displayed metabolic phenotypes compatible with each abundance-growth point, computed based on flux ranges for each reaction. These ranges are seen as descriptors of the system’s plasticity (35, 36) and allow us to distinguish between reactions that present flux plasticity, where their fluxes remain positive (i.e., always active) despite flux variations, from reactions associated with structural plasticity that shows zero flux on some solutions, emphasizing their putative replacement by alternative pathways. Moreover, the proposed framework allows projecting the metabolic phenotype in a lower dimensional space for interpretation, thus providing a practical exploration of metabolic phenotypes in a community characterized by different relative abundances and growing at suboptimal growth rates.

For illustration, the method is applied to a synthetic *E. coli* community with a mandatory cross-feeding of leucine and lysine, and an environmental community composed of *Acidithiobacillus ferrooxidans* Wenelen and *Sulfobacillus thermosulfidooxidans* Cutipay, where only the latter consumes organic matter disposed of by the community.

In both examples, the study of the abundance-growth space allows us to pinpoint how critical reactions respond to shifts in the environment, showing where changes in community plasticity occur. Notably, the method not only allows us to confirm that plasticity increases as the growth rate decreases as expected (29) but also highlights the relevance of the relative abundance of its members in the loss or gain of plasticity. In a more quantitative perspective, in both examples we show that despite the different degrees of plasticity, there are strong couplings between key reactions when reaching high growth rates or attaining very unbalanced communities.

## RESULTS

### Rationale of the abundance-growth space

We aim to understand mechanisms that support community structure and the ecosystem’s resilience to perturbations. To achieve this, we propose to study the metabolic phenotypes of a community through the definition of what we call the abundance-growth space.

A community model is constructed using single-organism genome-scale metabolic models, considering each organism as a compartment. Exchanges between organisms occur in the pool compartment. We assume the community is stable over time, i.e., all organisms grow at the same rate to maintain constant relative abundances, which is common in community modeling (13, 15, 16). The requirement of an identical growth rate between all organisms applies not at every time point but averaged over time. Thus, this hypothesis is a reasonable approximation for communities where composition is mainly maintained between spaced time points (15).

In this model, μ is the community growth rate, and the relative abundance $f_{i}$ of organism i appears as a factor of the flux bounds for any reaction of its respective organism (Materials and Methods). Thus, variations on the vector of abundances $F=\left(f_{1},\ldots ,f_{N}\right)$ and on μ affect the set of flux distributions that are feasible solutions of the model. Specifically, for a given abundance-growth pair (F,μ) , the *polytope* $P_{F,\mu }$ is defined as the set of all flux distributions satisfying model’s constraints. We define the abundance-growth space as the set of all feasible pairs (F,μ) , that is, when $P_{F,\mu }\neq \emptyset$ . We believe that this space is a good asset to capture the metabolic phenotype when changing growth rates and abundances of members of a community.

**Fig 1:**
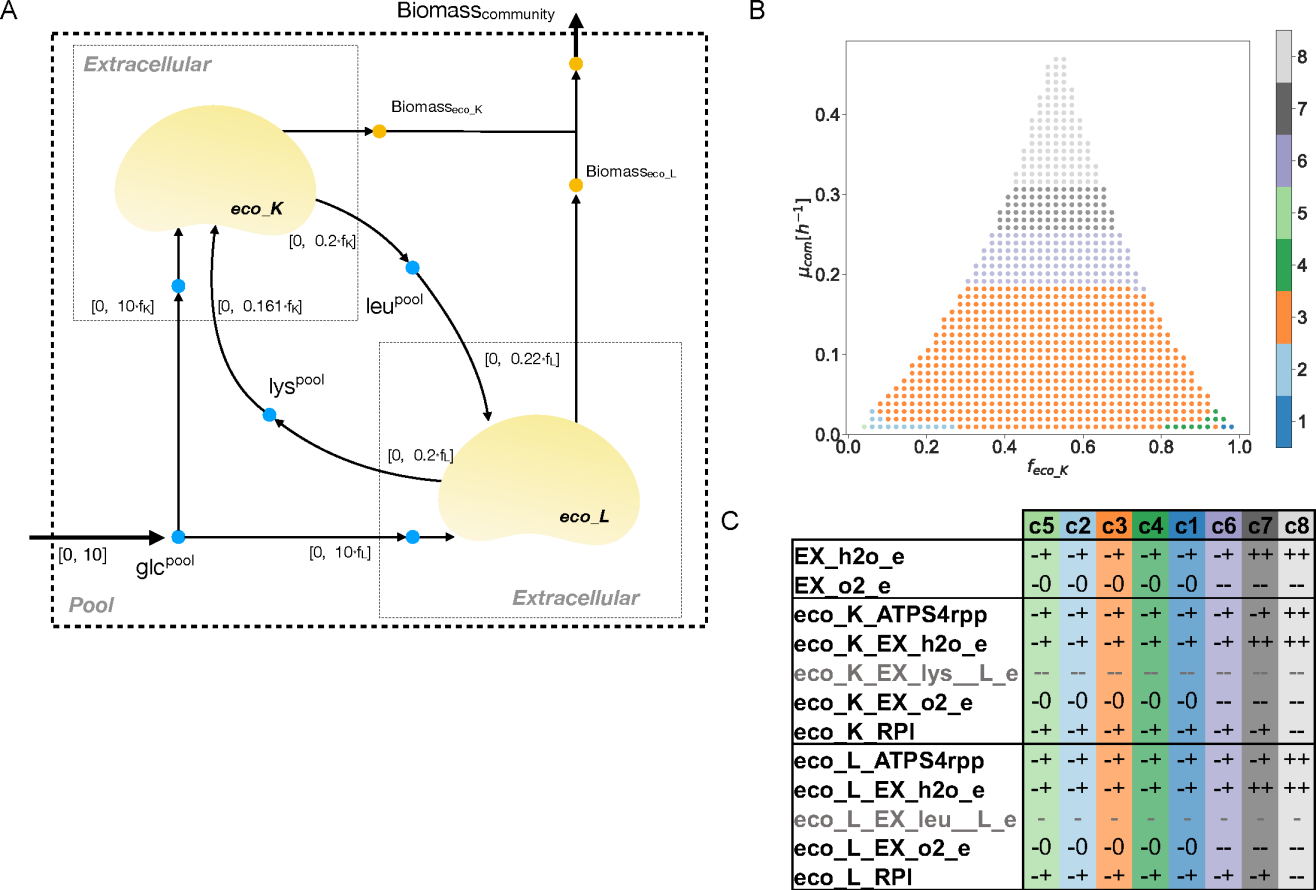
Analysis of the abundance-growth space for a synthetic *Escherichia coli* community. (**A**) A synthetic cooperative microbial community of two *E. coli* mutants is constructed by knocking out key genes for leucine (eco_L) and lysine (eco_K) production. Bounds on exchange reactions for each strain are constrained based on their abundances (depicted as intervals). (**B**) Cluster-partition ( k=8 ) of the abundance-growth space for the presented community with no leucine or lysine supplementation. (**C**) Table of reactions in which qualitative states change between neighboring clusters. Clusters are organized according to their location in the abundance-growth space from low to high growth rate and from *f*_ ${}_{e\,c\,o\,\_\,K}$_ 0 to 1. Lysine and leucine exchange reactions (gray rows) do not exhibit changes among the analyzed clusters. EX: exchange reactions, h2o: water, o2: oxygen, lys: lysine, leu: leucine, ATPS4rpp: ATP synthase, RPI: ribose-5-phosphate isomerase.

Characterizing $P_{F,\mu }$ for each point, (F,μ) of the abundance-growth space is computationally hard and complex to describe. Thus, we represent it by the minimum [ ${min}_{F,\mu }(r)$ ] and maximum [ ${max}_{F,\mu }(r)$ ] values of the flux $v_{r}$ of reaction r in $P_{F,\mu }$ . We propose the range between ${min}_{F,\mu }(r)$ and ${max}_{F,\mu }(r)$ as an indicator of metabolic plasticity as described in references 35 and 36. Indeed, a reaction r has flux plasticity if ${min}_{F,\mu }(r)\neq {max}_{F,\mu }(r)$ . In addition, the reaction has structural plasticity if zero is in between these extreme values, meaning that alternative pathways could replace r . To obtain a more quantitative description of $P_{F,\mu }$ , one can consider the set of combined feasible fluxes of pairs of selected reactions (quantitative coupling). Computationally, this representation is achieved by defining a grid ℱ of feasible abundance-growth points (Materials and Methods). For an example of the abundance-growth space for a community of two organisms, see Fig. 1B [we only plot $\left(f_{1},\mu \right)$ since $f_{2}=1-f_{1}$ ].

Following this rationale, for studying metabolic phenotypes given by a set of reactions of interest $R^{\prime }$ on an abundance-growth point (F,μ) , we assign a categorical value to $r\in R^{\prime }$ according to Table 1. In an exploratory study, $R^{\prime }$ can be the whole set of reactions, but in a more precise context, for instance when focusing on the community’s metabolic interactions, it can be restricted to exchange reactions. Then, for each point of the grid, we compute a categorical vector for reactions in $R^{\prime }$ , generating a partition of the abundance-growth space in zones with identical vectors. Depending on the size of $R^{\prime }$ , the number of zones can be huge. To overcome this, we define a cluster-partition, whose zones are obtained by a clustering algorithm, and we attribute a consensus categorical vector to each cluster-partition (Materials and Methods).

**TABLE 1:** Categorical values for a reaction r and a point (F,μ) of the abundance-growth space

Condition	Category	Flux sign	Flux variability	Flux requirement	Level of plasticity
${min}_{F,\mu }(r)={max}_{F,\mu }(r)=0$	0	Zero	Fixed	Off	No plasticity
${min}_{F,\mu }(r)={max}_{F,\mu }(r)<0$	−	Negative	Fixed	Mandatory	No plasticity
$0<{min}_{F,\mu }(r)={max}_{F,\mu }(r)$	+	Positive	Fixed	Mandatory	No plasticity
${min}_{F,\mu }(r)<{max}_{F,\mu }(r)<0$	−−	Negative	Variable	Mandatory	Flux plasticity
$0<{min}_{F,\mu }(r)<{max}_{F,\mu }(r)$	++	Positive	Variable	Mandatory	Flux plasticity
${min}_{F,\mu }(r)<{max}_{F,\mu }(r)=0$	−0	Negative or zero	Variable	Optional	Structural plasticity
$0={min}_{F,\mu }(r)<{max}_{F,\mu }(r)$	0+	Positive or zero	Variable	Optional	Structural plasticity
${min}_{F,\mu }(r)<0<{max}_{F,\mu }(r)$	−+	Any	Variable	Optional	Structural plasticity

### The abundance-growth space for a synthetic *E. coli* community

To illustrate this method, we first consider a synthetic cooperative community composed of two *E. coli* strains auxotrophic for leucine (eco_L) and lysine (eco_K) (File S1, https://github.com/mathomics/ecosystem), studied in reference 37. Consumption and production of these amino acids were constrained based on their reported fluxes (Materials and Methods). In this community, cross-feeding of leucine and lysine is critical for survival (Fig. 1).

We obtain an abundance-growth space whose shape resembles a concave triangle (Fig. 1B). Its dimensions are influenced by changes in the production capacity of leucine and lysine, as well as its external supplementation (Fig. S1), as discussed below. This shape shows that at high growth rates, abundances need to be balanced ( $f_{e\,c\,o\,\_\,K}∼f_{e\,c\,o\,\_\,L}$ ), which is consistent with reference 37. On the other hand, communities with a dominant strain can only grow at lower rates, which was attributed to the less abundant strain being unable to satisfy amino acid requirements from the other (37). Indeed, at a fixed abundance of eco_K (eco_L), the minimum flux value for the production of leucine (lysine) reaches the upper bound in the model, making it unfeasible for greater growth of the community (Fig. S2).

From the 5,068 reactions of the model, a cluster-partition is performed. Eight clusters allow us to distinguish relevant changes in this space (Materials and Methods and Fig. S3a). We distinguish 116 reactions whose consensus values change between clusters, 1,880 that are unable to carry any flux in the abundance-growth space, and 2,371 presenting fixed non-zero flux values (File S2, https://github.com/mathomics/ecosystem). Blocked reactions are associated with the exchange of metabolites absent in the environment of the simulated community, as well as consequences of gene knockout performed on both strains. Fixed reactions are associated with biomass production requirements, such as NAD and tetrahydrofolate synthesis and transport of ions present in biomass. Interestingly, only leucine consumption by eco_L exhibits a fixed flux value in all the abundance-growth space (status –), while eco_K displays a small range in which consumption of lysine is observed (status –).

From the 116 changing reactions, 98 appear only in shifts between clusters of unbalanced communities at very low growth rates (clusters 2, 5, 1, and 4). Among the remaining 18 reactions, we determine that 10 are able to describe all the transitions between clusters, since redundant reactions (such as transporters of O_2_ and H_2_O) can be removed. Changes of these 10 reactions show that the community is set toward cell growth by breaking down glucose, rather than amino acid synthesis and consumption. Indeed, these shifts correspond to indirect measurements of how active glucose catabolism is, hence displaying exchanges for oxygen, carbon dioxide, and water, for the community. Between clusters 7 and 8, shifts are observed in reactions depicting an active metabolism toward biomass synthesis: ATP synthesis (from −+ to + [Fig. 1C]) and ribose-5-phosphate isomerase (RPI), which reverses its flux toward nucleotide synthesis.

In addition, to get a deterministic description of the abundance-growth space, we compute the partition given by the qualitative vectors associated with these 10 reactions. This yields nine areas (Fig. S4) revealing in more detail the shifts observed in the cluster partition. Particularly, shifts forcing ATP synthesis and water exchange by eco_L (Fig. 1A).

**Fig 2:**
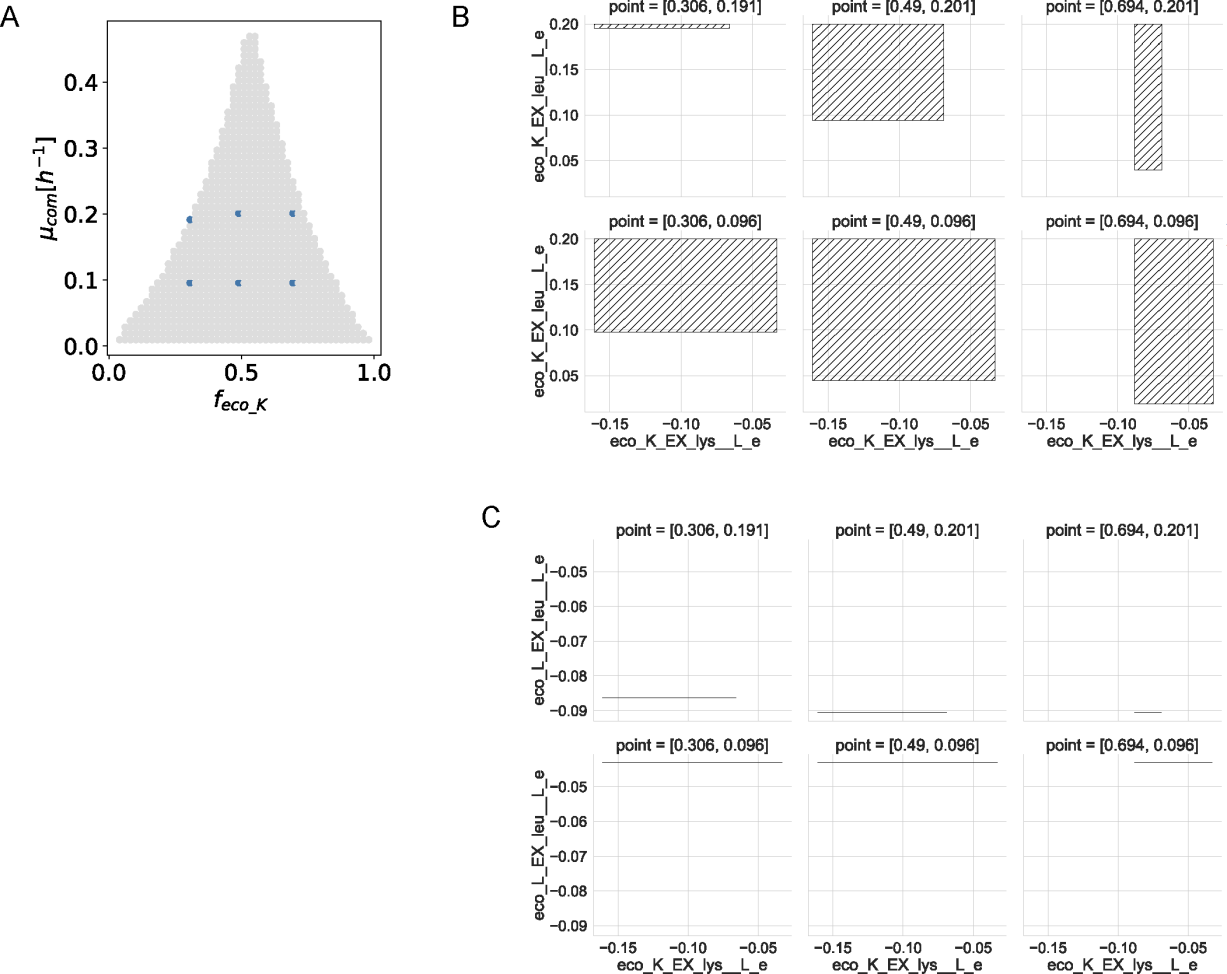
Quantitative flux coupling analysis for the exchange of lysine and leucine in the synthetic *E. coli* community. (**A**) Selected points in the abundance-growth space. (**B**) Feasible fluxes for the consumption of lysine and production of leucine by eco_K at the different points marked in panel **A**. (**C**) Feasible fluxes for the consumption of lysine by eco_K and consumption of leucine by eco_L. Fluxes are in units of (mmol/g DW_org_/h).

Finally, since states for both leucine and lysine exchanges remain constant along the abundance-growth space, we study the degree of quantitative coupling between these reactions. For this, we plot flux values for different couples of these reactions in six points of the space (Fig. 2A). In particular, Fig. 2B shows the consumption of lysine and production of leucine by eco_K. Here, flux ranges are affected by both composition and growth in such a way that when eco_K is more abundant and requires higher growth rates, lysine consumption by this bacterium tends to be fixed, but the production of leucine can vary on a wide interval. On the contrary, leucine production by eco_K tends to be fixed when this bacterium is less abundant at higher growth rates.

Also, in Fig. 2C, consumption of lysine by eco_K vs consumption of leucine by eco_L are plotted, showing the following assymetry: eco_L requires a fixed flux of leucine that increases at higher growth; on the other hand, the flux of lysine consumption by eco_K is more flexible, living in an interval which decreases with its abundance. Fixed values on leucine consumption reflect its exclusive requirement for biomass synthesis, while lysine can be transformed into 1,2-diaminepentane through the lysine decarboxylase reaction and this metabolite can be exported.

**Fig 3:**
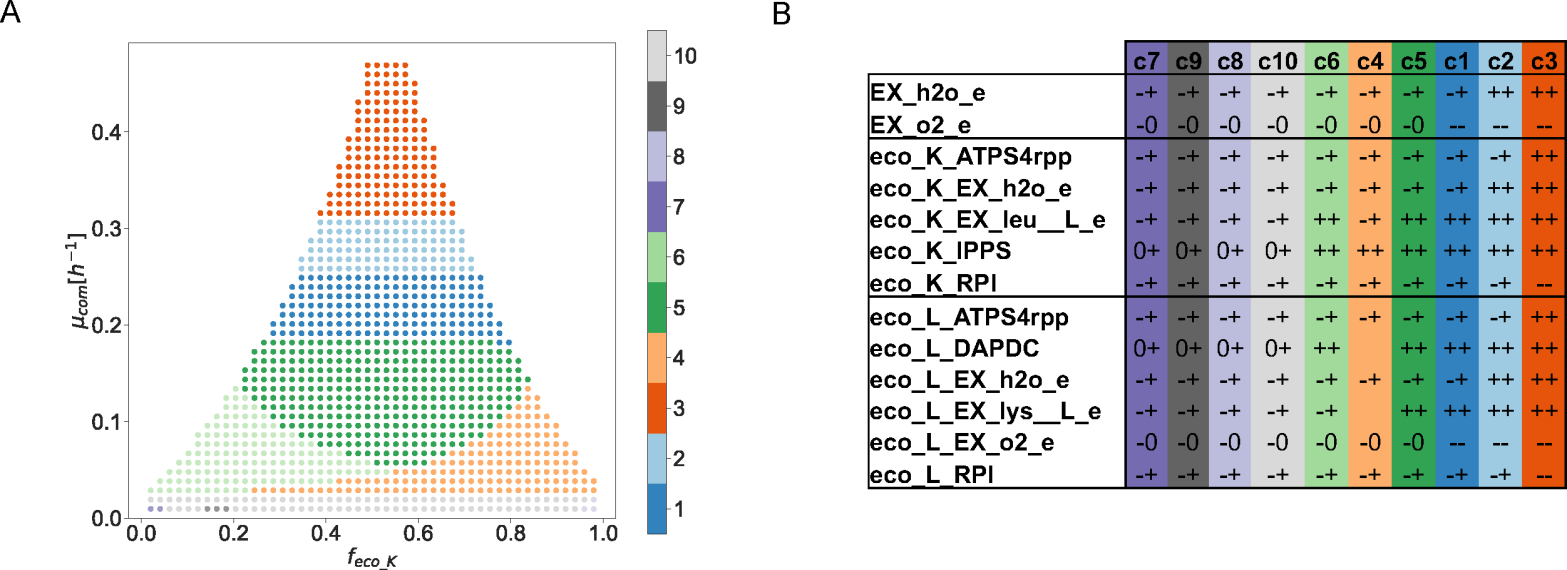
Analysis of the abundance-growth space for a synthetic community of *E. coli* supplemented with leucine and lysine. (**A**) Cluster-partition ( k=10 ) of the abundance-growth space. (**B**) Key reactions associated with changes in their states between neighboring clusters together with their qualitative values along the clusters. Clusters are organized according to their location in the abundance-growth space from low to high growth rates and from *f*_ ${}_{eco\_K}$ _0 to 1. EX: exchange reactions, h2o: water, o2: oxygen, lys: lysine, leu: leucine, ATPS4rpp: ATP synthase, IPPS: 2-isopropylmalate synthase, RPI: ribose-5-phosphate isomerase, DAPDC: diaminopimelate decarboxylase

Although this has not been reported (37), by simulating the supplementation of leucine and lysine, this method can give insights into how metabolism is affected by shifts in its environment. When both lysine and leucine are supplemented (Fig. 3) at a flux of 0.01 (mmol/g DWcom/h), we get that a cluster-partition of 10 sets accurately represent qualitative states (Fig. S3B).

We observe an area of low growth rates, where the community can opt to not synthesize leucine and lysine (clusters 7, 9, 8, and 10). By increasing the community growth rate, both bacteria are required to produce leucine and lysine. First, in clusters 4 and 6, the key reaction for leucine synthesis (eco_K_IPPS) changes from 0+ to ++. But its supplementation for the other bacterium (eco_K_EX_leu_L_e) is only required when eco_K is less abundant (cluster 6) (Fig. 3). In Fig. S5, a partition of the abundance-growth space for the previous reactions highlights these phenomena. For lysine synthesis, an analogous phenomena is observed, where its key reaction (eco_L_DAPDC) shifts from 0+ to ++. From this point upwards (clusters 5, 1, 2, and 3), both bacteria are required to supplement lysine and leucine, respectively (++ state). No more changes are observed with respect to the scenario without the supplementation of lysine and leucine.

The quantitative analysis of Fig. 2 in this scenario only reflects that there is more plasticity due to amino acid supplementation (Fig. S6), measured as an increase in the range of minimum and maximum fluxes.

### The abundance-growth space for a bioleaching community

To test the method in an environmental community, we consider a simple microbial consortium between *A. ferrooxidans* Wenelen and *S. thermosulfidooxidans* Cutipay (see File S3 and File S4 in https://github.com/mathomics/ecosystem and Materials and Methods), obtaining a community competing for inorganic energy sources (Fe(II) and thiosulfate). Both bacteria are able to fix carbon available as CO_ ${}_{2}$_ but only Cutipay can consume organic matter made available by the degradation of community biomass, which is modeled as a pseudo-reaction transforming a fraction α of the biomass produced by the community (Materials and Methods).

We consider the case study where α=0.2 and a substrate level composed of energetically equivalent amounts of Fe(II) and thiosulfate. The obtained abundance-growth space has a convex shape such that the maximum community growth rate increases when Wenelen decreases, showing that Cutipay is more efficient in producing biomass than Wenelen. Since both bacteria have been observed together in equilibrium (38), it seems that they are not maximizing the growth rate of the community in these conditions.

**Fig 4:**
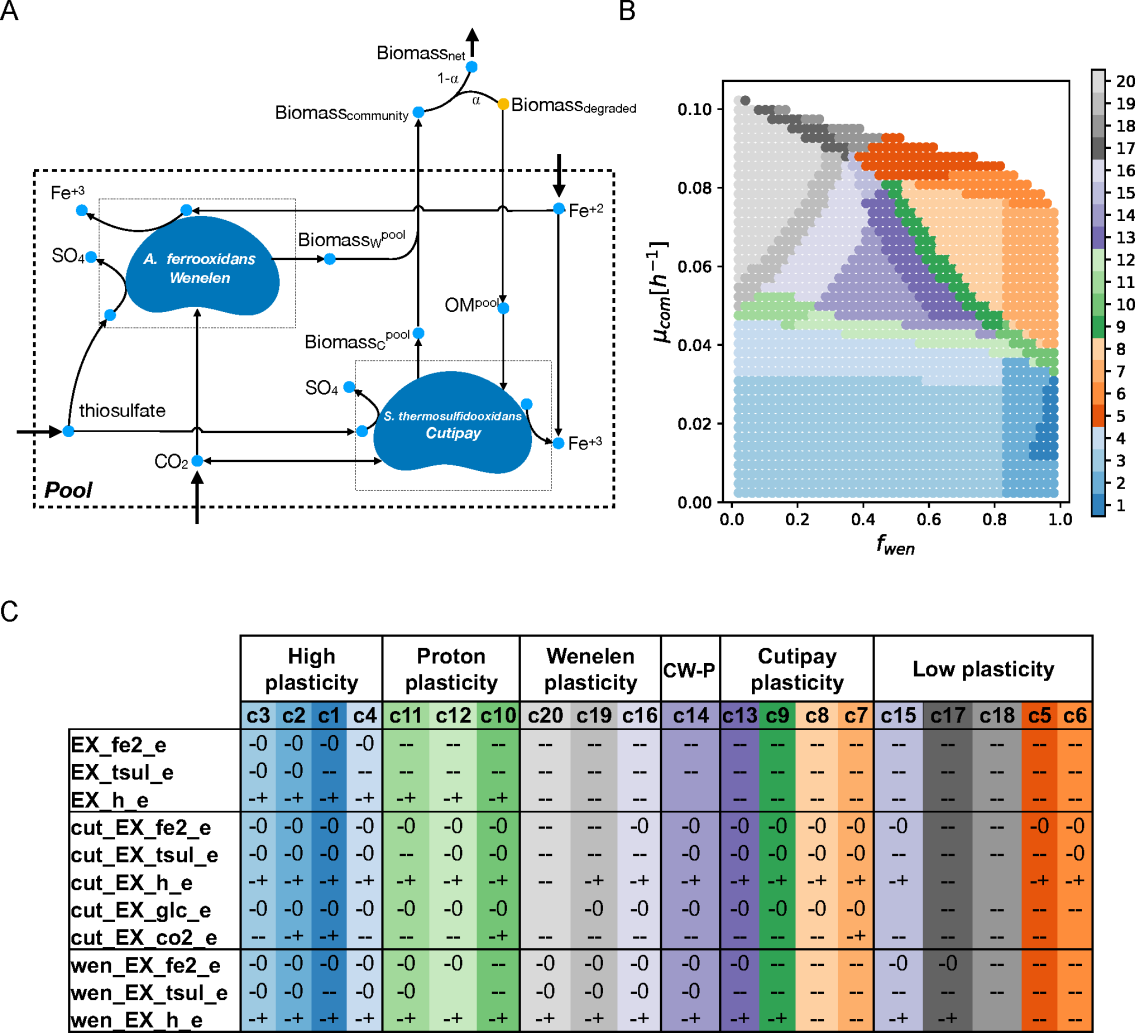
Analysis of the abundance-growth space for the bioleaching community. (**A**) A community composed of *A. ferrooxidans* Wenelen (wen) and *S. thermosulfidooxidans* Cutipay (cut) is studied in an environment with Fe(II) and thiosulfate in the presence of organic matter. A pseudo-reaction represents organic matter (OM) availability as a fraction ( α , here α=0.2 ) of the total community biomass. (**B**) Twenty clusters are computed for the 100 exchange reactions of the community model. (**C**) Key reactions associated with qualitative changes between neighboring clusters define six areas for the behavior of this community. A status is associated with a cluster when over 80 % of the points in the cluster exhibit a certain state. If no consensus is reached, an empty cell is presented. Clusters are organized according to their location in the abundance-growth space from low to high growth rate and from *f*_ ${}_{wen}$_ 0 to 1. OM: organic matter, EX: exchange reactions, h: hydrogen (proton), tsul: thiosulfate, co2: carbon dioxide, fe2: Fe(II), glc: organic matter, CW-P: Cutipay Wenelen plasticity.

Considering $R^{\prime }$ as the set of 100 exchange reactions, a cluster-partition is computed using 20 clusters (Fig. 4B) (Materials and Methods and Fig. S7 to justify the number of clusters).

We find that 23 reactions change between neighboring clusters (Fig. 4B). The 27 remaining reactions are blocked, coupled with their biomass production requirements, or exhibited structural plasticity on each grid point. The curation of these 23 reactions allowed to remove those that are coupled in all clusters, such as consumption of Fe(II) and production of Fe(III), pairs of exchange reactions for H_ ${}_{2}$ _O and H, and SO_ ${}_{4}$_ and H. Additionally, reactions in which changes are only attributed to a few clusters were removed, notably the exchange of H_ ${}_{2}$ _, which production is optional only in cluster 1. After this curation, 11 critical reactions associated with iron, sulfur, and carbon metabolism were obtained. Five of them belong to Cutipay, three to Wenelen, and three to the microbial community as a whole (Fig. 4C).

Six sections are distinguished (Fig. 4B and C). A high plasticity section is defined at lower growth rates, where most exchanges are optional (−0 and −+) and independent of abundances. As growth requirements increase, oxidation of both inorganic energy sources by the community becomes mandatory (–), as well as the consumption of sulfur and iron, which indicates a loosing of global structural plasticity. Next, we define a proton plasticity section (clusters 10, 11, and 12), where the community needs to consume Fe(II) and thiosulfate, preserving structural plasticity (−+) for the exchange of protons (H). This indirectly measures the balance between Fe(II) and thiosulfate oxidation, since the first requires protons while the second produces them (39). Thus, in this section, the community can still opt not to affect the pH of its environment.

In the Wenelen plasticity section, all reactions associated with this bacterium exhibit structural plasticity. Here Cutipay shifts from no plasticity for Fe(II) oxidation in cluster 20 (where it is abundant) to structural plasticity in cluster 16. Also, we observe a less active Fe(II) oxidation (cut_EX_h_e, from – to −+). Analogously, in the Cutipay plasticity section its associated reactions display structural plasticity, while Wenelen loses it for thiosulfate (cluster 13) and iron (cluster 9) consumption when approaching $f_{wen}=1$ . Cluster 7 shows an increase in carbon metabolism structural plasticity, where it no longer requires CO_ ${}_{2}$_ consumption to support its growth (cut_EX_co2_e).

In between the above two sections stands the Cutipay-Wenelen plasticity section, where high plasticity is observed for both organisms. It is a desirable area where organisms can adapt to shifts in their environment, maintaining high growth rates. Finally, in the low plasticity section, characterized by the highest growth rates, all reactions lose plasticity, and the high energy requirements force Cutipay to consume organic matter (represented by equivalent carbon units in the form of glucose [Materials and Methods]).

Eleven critical exchange reactions were selected from the previous clustering analysis, producing a partition of 40 areas, among which an organic matter consumption area is clearly defined for Cutipay as well as a much specific description of the low plasticity section (Fig. S8), thus confirming in a deterministic way previous observations regarding plasticity in the abundance-growth space.

**Fig 5:**
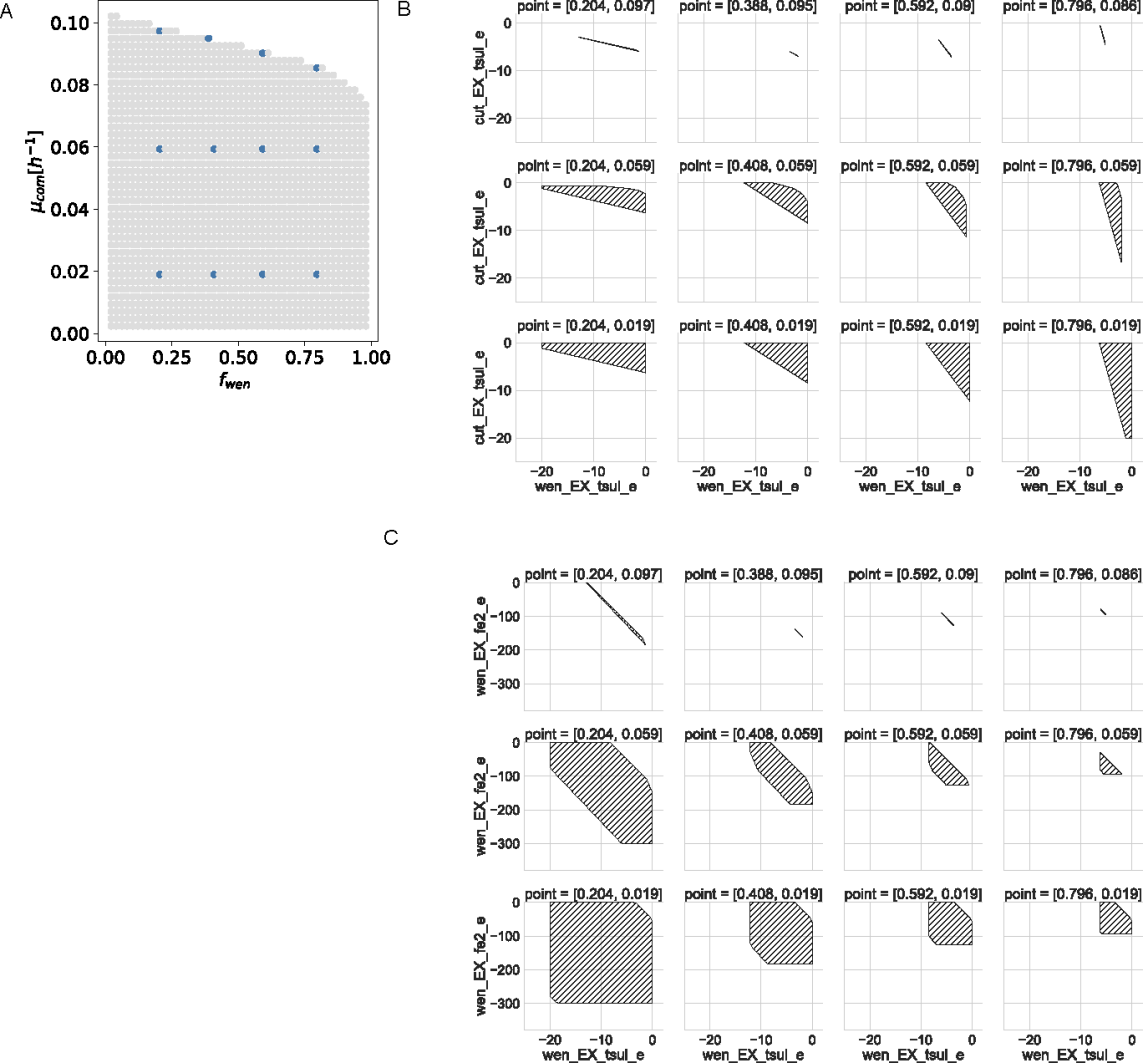
Quantitative flux coupling analysis for competition for thiosulfate and distribution of resources by *A. ferrooxidans* in the bioleaching community. (**A**) Selected points in the abundance-growth space. (**B**) Feasible fluxes for thiosulfate exchanges (EX_tsul_e) for *A. ferrooxidans* (wen) and *S. thermosulfidooxidans* (cut) at the different points marked in panel **A**. (**C**) Feasible fluxes of Fe(II) and thiosulfate exchanges (EX_fe2_e and EX_tsul_e) for *A. ferrooxidans* at different points marked in panel **A**. Fluxes are in units of (mmol/g DW_ ${}_{org}$ _/h).

To quantitatively illustrate previous results, explore competition for resources (Fig. 5B; Fig. S9), and use of different substrates to support cell growth (Fig. 5C; Fig. S10), we computed the feasible solution space for critical pairs of reactions associated with the consumption of energy sources at specific points of the abundance-growth space. As expected, there is an overall reduction in plasticity while moving into the growth-oriented area, where exchange fluxes for energy sources are perfectly coupled. This result is evident in the points (0.388 and 0.095), where for a given consumption of thiosulfate, there is a unique consumption of iron (Fig. 5B). Plasticity reduction is also observed when moving into extreme compositions of the community, where being more abundant results in having less specific consumption rates. Notably, when the community becomes balanced, the feasible solution space of critical pairs of reactions concentrates more at higher growth rates. This is less evident just from the abundance-growth space observation.

#### Impact of organic matter availability on a bioleaching community

Organic compounds are detrimental for some autotrophic bioleaching bacteria (40), toxic for chemolithotrophic (41), having inhibitory effects in iron oxidation (42), thus favoring the abundance of heterotrophs or facultative heterotrophs in bioleaching communities (43).

**Fig 6:**
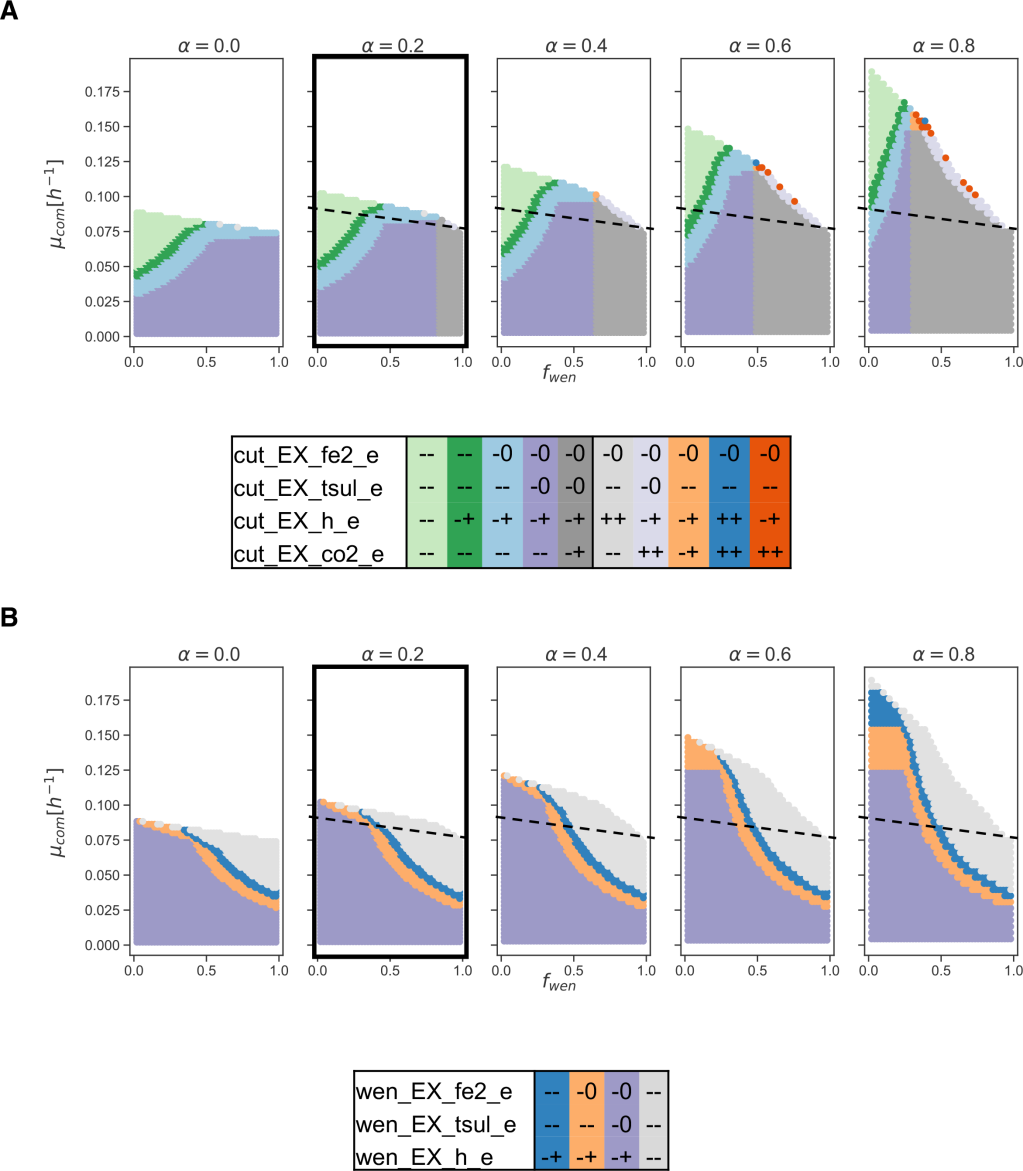
Effects of organic matter availability in the bioleaching community. A partition of the abundance-growth space is computed for key reactions of the bioleaching community when increasing organic matter availability ( α ). α represents the ratio of the biomass generated by the community that could be degraded to serve as an additional carbon source. The reference case is highlighted with thicker edges; dashed lines show the maximum community growth rate for α=0 . (**A**) Qualitative states of reactions of *S. thermosulfidooxidans* Cutipay (cut). (**B**) Qualitative states of reactions of *A. ferrooxidans* Wenelen (wen). Partition tables are ordered according to the location of different zones in the abundance-growth space from lower to higher growth rates, from *f*_ ${}_{wen}$_ 0 to 1, and their occurrence in different scenarios from left to right. EX: exchange reactions, fe2: Fe(II), tsul: thiosulfate, h: hydrogen (proton).

Scenarios with increasing amounts of organic matter availability were simulated by moving α , and partitions of the abundance-growth space were computed for essential bioleaching reactions (Fig. 6). By increasing α , a direct effect in the enlargement of the space with the maximum growth rate of the community and an increase of the higher structural plasticity areas are observed (Fig. 6). In Wenelen, this only happens until the maximum uptake of inorganic sources is reached when α=0.6 . Interestingly, in Cutipay, the enlargement of the space is given by the mandatory consumption of organic matter (− −), and it exhibits a shift where CO_ ${}_{2}$_ consumption is no longer required to support its growth (gray area in Fig. 6A). Moreover, new areas appeared near the maximum community growth section, where this bacterium is forced to break down organic matter, hence producing CO_ ${}_{2}$_ to survive (Fig. 6). This shows that organic matter is beneficial for achieving higher growth rates and that Cutipay does not present a preference for organic matter over inorganic sources.

This result, at higher growth rates, initially contradicts literature regarding the behavior of bioleaching communities in the presence of organic matter, which is characterized by an increased abundance of heterotrophs and facultative heterotrophs, which favor the consumption of carbon sources for energy production (43) and will be explored by imposing additional objectives. This could mean that these communities grow at low rates.

#### Impact of substrate composition on the bioleaching community

Different stages of the bioleaching process are characterized by changes in chemical and physical properties, which shape the distribution of resources, metabolic states, and community compositions (43). In particular, uniform distribution of substrates is not always guaranteed, and nutrient bioavailability could pose a bottleneck for bioleaching efficiency.

**Fig 7:**
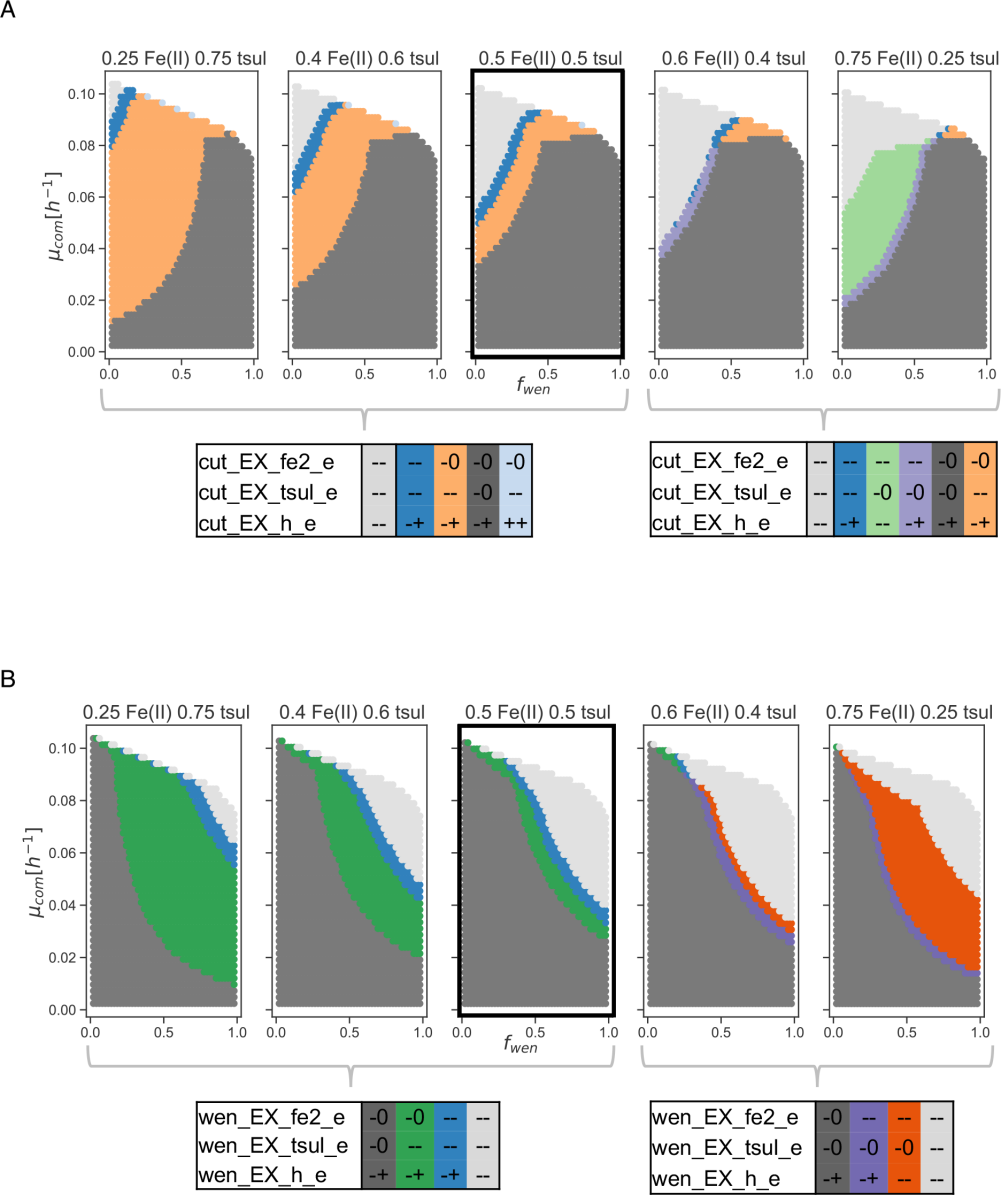
Effect of relative substrate availability in the bioleaching community. A partition of the abundance-growth space defined by qualitative states for key reactions of the bioleaching community in scenarios with changing ratios between both energy sources. Two partition tables for each bacteria are displayed for the qualitative states in different scenarios. (**A**) Partition tables of reactions for *S. thermosulfidooxidans* Cutipay. (**B**) Partition tables of reactions for *A. ferrooxidans* Wenelen. The reference case is highlighted with thicker edges. Partition tables are ordered according to the location of different zones in the abundance-growth space from lower to higher growth rates, from *f*_ ${}_{wen}$_ 0 to 1 and from their occurrence in different scenarios from left to right.

Consistently, in our model, changing the relative availability of Fe(II) and thiosulfate has drastic effects on the behavior of the community (Fig. 7). Iron-predominant scenarios present new metabolic states where iron availability overpasses the effect of preference for thiosulfate as an energy source, which has been observed up to this point in these analyses. It is worth noticing that sulfur compounds have higher energy yields than Fe(II) since they have more electrons available (44). Although, *A. ferrooxidans* is believed to exhibit a preference for iron (45), evidence shows the simultaneous expression of genes for the consumption of iron and sulfur (46), being solubilization of sulfur compounds is key to consuming both energy sources (47). This result is consistent with our analysis, where a preference for Fe(II) oxidation is only displayed when its availability surpasses thiosulfate.

#### Impact of considering alternative objectives

**Fig 8:**
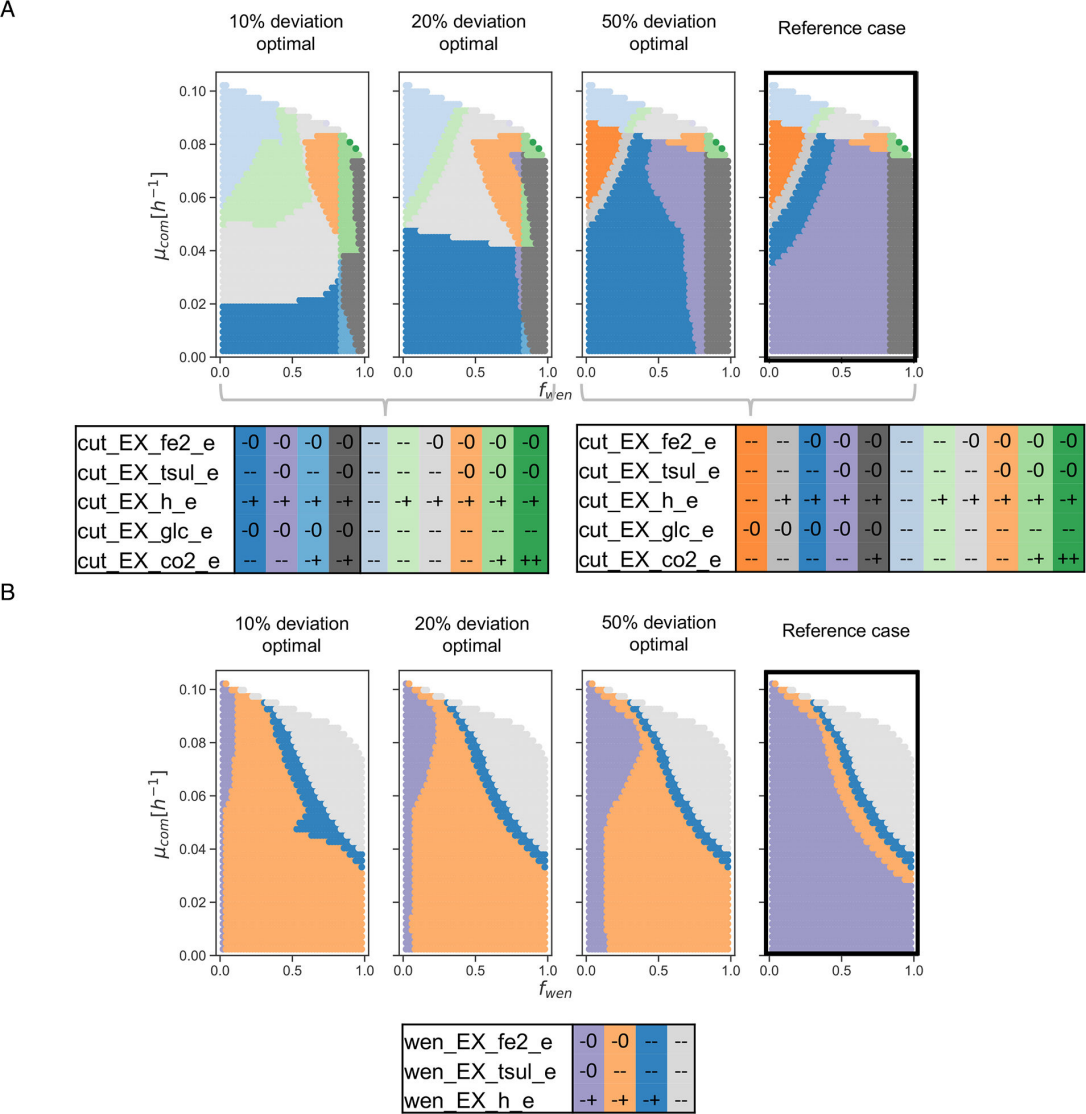
Effect on qualitative states when optimizing energetic efficiency in the bioleaching community. A partition of the abundance-growth space defined by qualitative states for key reactions of the bioleaching community in scenarios where 10%, 20%, and 50% of deviation from the optimal solution for energy efficiency is allowed. (**A**) Two partition tables are displayed for *S. thermosulfidooxidans* Cutipay (cut) for the qualitative states presented in different scenarios (**B**). Partition table of reactions for *A. ferrooxidans* Wenelen (wen). Partition tables are ordered according to the location of different zones in the abundance-growth space from lower to higher growth rates, from *f*_* ${}_{wen}$*_ 0 to 1, and from their occurrence in different scenarios from left to right.

For testing a more realistic representation of a bioleaching community, a parsimonious FBA, where minimization of the sum of fluxes is performed as a proxy for energetic efficiency. This has surged as a realistic alternative to retrieve flux distributions (29, 48). This analysis allows a 10%, 20%, and 50% deviation from the optimal solution to maintain a certain degree of plasticity. The results (Fig. 8) show a significant change with the one observed in Fig. S8.

Regarding energy source preferences, it is remarkable how thiosulfate becomes mandatory at significantly lower growth rates as we are close to the optimal energy efficiency value. However, the degrees of plasticity in Fe(II) consumption remain unaltered concerning the reference case in both bacteria. This observation is valid in all areas of the space, except for Cutipay in $f_{wen}\geq 0.8$ , where this bacteria retains structural plasticity for both inorganic sources. Interestingly, organic matter consumption by this bacteria is required at lower growth rates, which is in agreement with the literature, where the presence of organic matter is beneficial for heterotrophs that use this energy source for biomass production (43, 49, 50).

## DISCUSSION

Environmental communities are subjected to constant shifts that affect their survival. Adaptation to such changes includes gene mutations (51), gene transfer, and even gene loss in collaborative communities (52), as well as the development of regulatory processes that result in efficient resource distribution such as flux distributions that result on increased metabolic plasticity (30, 53). This raises the long-standing discussion of whether the community **opts** to live at suboptimal growth rates to increase the capacity to use alternative metabolic pathways and if it is possible to predict their metabolic phenotypes at a given condition, as proposed in references 29 and 30.

Constraint-based modeling for single organisms has historically based most of their flux predictions on the maximization of biomass production (54). This assumption yields promising results for organisms well characterized in laboratory settings for being efficient in the growth and synthesis of a product of interest (55, 56). In contrast, organisms in nature are exposed to constant shifts in their environment, which implies the production of secondary metabolites that provide favorable conditions for survival, deviating resources to the detriment of growth maximization (5, 29). Additionally, communities interact in a complex manner (51, 52), where the abundance of each member has implications in the growth rate of the community.

Several optimization strategies have been developed to characterize interactions between organisms (12 – 15). From a more ecological perspective, the notion of metabolic niche was proposed to characterize all possible environments in which an organism could live in suboptimal conditions (33). This niche concept uses the feasible flux space to study how organisms interact with their environment, represented by few exchange reactions, depicting new relationships between genotype and metabolic phenotypes (33). Even though these methods consider all feasible metabolic fluxes, they do not fully explore emergent properties arising from organism relative abundances.

In this work, we introduce the abundance-growth space defined by the composition of a community and its growth rate, as an approach to characterize the metabolic phenotype attained by a community and the plasticity of its metabolism. More precisely, we describe, on each point of this space, which reactions are required to carry flux (mandatory vs optional flux) and whether their function can be partially replaced by alternative pathways (variable vs fixed flux). Since these characteristics vary across the abundance-growth space, partitioning this space into zones of similar metabolic profiles gives a characterization of the most relevant phenotypes presented and their relation with the composition of the community.

In addition, the partition of the abundance-growth space allows us to pinpoint critical reactions involved in significant changes in interactions of the community with their environment and establishes, in a precise manner, where critical changes in community plasticity occur. Notably, in both examples developed here, this method allows to unveil how plasticity changes in the abundance-growth space, not only confirming that plasticity increases as the growth rate decreases as expected (29) but also highlighting the relevance of the relative abundance of its members in the loss/gain of plasticity and in the flux coupling of some reactions. Additionally, in these examples only few reactions are found to explain most of the metabolic shifts and many reactions become fixed. Particularly, reactions related to inorganic energy sources exchange and organic matter consumption in the bioleaching community, and reactions for leucine and lysine exchange and energetic metabolism in the synthetic *E. coli* community are critical for community plasticity. Certainly, this number can increase as the environment becomes more complex.

However, the characterization of reactions solely based on their qualitative values does not allow to recover the degree of coupling between reactions. This is complemented by performing a quantitative flux coupling analysis, which illustrates how the coupling between reactions changes along the abundance-growth space. In both examples, we show that despite the different degrees of plasticity, there are strong couplings when reaching high growth rates or attaining very unbalanced communities.

The examples presented show the versatility of the method to study changes in the metabolic phenotype in communities with different interactions. On the one hand, those that supply each other with metabolites (cross-feeding) and on the other hand, communities where their complexity lies in variable capacities of its members to consume energy sources, such as the bioleaching community where autotroph and heterotroph bacteria coexist. Interestingly, the abundance-growth space shows a very different behavior in different cases.

An obvious observation of the method has to do with its scalability when the size of the community grow, since the number of grid points can increase exponentially. It is worth mentioning that, both the computation of the grid points in the abundance-growth space and the categorical values of the reactions associated to each feasible grid point admit a parallel implementation, which makes this method usable for communities of few organisms. However, for bigger communities, the method it is still versatile. For instance, one could compute the abundance-growth space for selected combinations of two or three organisms, fixing the abundance of the remaining members of the community, at a reasonably computing cost and still keeping a suitable visualization, or one could reduce this analysis to a fixed number of interesting points of the abundance-growth space.

In any case, we think this method provides a valuable tool for studying, in a detailed manner, different metabolic scenarios where a small community decides to live given a certain environmental condition and to reveal the critical drivers for such functioning, considering the relevance of their relative abundances. In addition, we believe that it could provide a useful approach for the design of synthetic communities of high impact in biotechnology and medicine.

## MATERIALS AND METHODS

### Modeling microbial community metabolism

An expansion of constraint-based modeling for microbial communities was proposed by Koch et al. (13). In summary, N organisms belonging to a community are considered. For each organism i∈{1,…,N} , a single model comprised a set of $M_{i}$ metabolites differentiated by organism and compartment and a set $R_{i}$ of all reactions of organism i . These reactions include all internal reactions, transport between compartments, and exchange of metabolites with their media. A biomass reaction $r_{b\,i\,o\,m\,\_\,i}$ is also included in $R_{i}$ , whose stoichiometric values on their substrates correspond to the amounts (in millimoles) of metabolites present in 1 g of dry weight of biomass of the organism i (g DW_i_).

If *v*_*r*_ denotes the flux over reaction r expressed in (mmol/gDW*_i_*/h), then the following constraints exist for organism i :


$${LB}_{r}\leq v_{r}\leq {UB}_{r},\text{ for all }r\in R_{i},$$

$$\begin{array}{rcl} & & \sum_{r\in R_{i}}S_{mr}v_{r}=0,\text{ for all }m\in M_{i}. \end{array}$$

The constants $LB_{r}$ and $UB_{r}$ are the lower and upper bounds for the flux over a reaction r and are used to control the amount and direction of fluxes in the system.

Given N single-organism models, a community is represented as a single compartmentalized system as previously established in references 16 and 57. In this model, each metabolic network is incorporated as a meta-compartment with an additional pool compartment that represents the shared media where metabolites can be exchanged among community members and the exterior.

A set of pool metabolites $M_{pool}$ is created containing metabolites present in the extracellular compartment of at least one organism. Consequently, any exchange reaction of organism i is considered a transport reaction between the extracellular compartment of i and the pool. Finally, a new set $R^{EX}$ of exchange reactions is defined to control external conditions for all metabolites in the pool.

In this community model, each member i makes up a fraction *f*_*i*_ of the total amount of community biomass (with $\sum_{i=1}^{N}f_{i}=1$ ). These fractions are relevant to compare the feasible fluxes on reactions of different organisms accurately.

Indeed, in this community model, fluxes are expressed in a single unit (mmol/g DW_com_/h), where g DW_com_ is a gram of dry weight of the community biomass. Since bounds on reaction fluxes of organism i were originally expressed in (mmol/g DW_*i*_/h) in the single-organism model, they must be recomputed in the community model, which is done by weighting by *f*_*i*_ each bound previously defined on a flux reaction of organism i (15).

Given this unit conversion, the flux $v_{biom\_i}$ of the biomass reaction of organism i is not expressing the growth $\mu_{i}$ of organism i , but the grams of organism i produced by each gram of the community per hour. Hence, if we consider a state of balanced growth, where the fraction *f*_*i*_ of each organism i is maintained over time, then we should impose that all organisms are growing at the same rate: $\mu_{1}=\ldots =\mu_{N}=\mu_{com}$ , which is equivalent to imposing: $v_{biom\_i}=f_{i}\;\mu_{com}$ for all i∈{1,…,N} (15).

The balanced growth assumptions indicate that the community has reached a stable state that allows the organisms to maintain their relative abundances over time, which is a common assumption in community modeling (13, 15, 16). If this constrain is not assumed, the faster-growing organism will ultimately displace all other organisms in the community. The requirement of identical growth rate between all organisms applies not necessarily at every time point but averaged over time, allowing small variations in the relative abundances. Thus, this hypothesis is a reasonable approximation for communities where composition is mainly maintained between spaced time points (15).

Considering all these observations, the set of constraints on the fluxes *v*_*r*_ of the community are the following:


$$\begin{array}{cc} \sum_{r\in R_{i}}\;\;S_{mr}v_{r}=0, & m\in M_{i},i\in 1,...,N, \end{array}\;\begin{array}{cc} V_{m\_pool\_exchange}=\sum_{i=1}^{N}V_{m\_extracell\_i\to pool}, & m\in M_{pool} \end{array}\;\begin{array}{cc} f_{i}LB_{r}\leq v_{r}\leq f_{i}UB_{r}, & r\in R_{i},i\in \{1,\ldots ,N\} \end{array}\;\begin{array}{cc} LB_{m\_pool\_exchange}\leq v_{m\_pool\_exchange}\leq UB_{m\_pool\_exchange}, & m\in M_{pool} \end{array}\;\begin{array}{c} \mu_{com}=\sum_{i=1}^{N}V_{bio\_i}, \end{array}\;\begin{array}{cc} v_{biom\_i}=f_{i}\mu_{com}, & i\in \{1,\ldots ,N\}. \end{array}$$

If the fractions *f*_*i*_ are fixed (as in cFBA [16]) or if the community growth rate $\mu_{c\,o\,m}$ is fixed (as in the case of SteadyCom [15] and RedCom [13]), then all constraints are linear.

Eventually, any other interaction between the organisms or additional information that can be expressed as a linear constraint on the fluxes can be easily added to the model. For instance, the model can directly include fixing values to reaction fluxes or imposing coupling between reactions. In the case of the bioleaching community, a pseudo-reaction models the capacity of one organism to use degraded organic matter as an additional energy source.

### Analysis of the abundance-growth space

The definition and analysis of the abundance-growth space are performed as follows.

*Definition of abundance-growth space*. Considering N organisms in the community, an abundance-growth pair is given by $(F,\mu )=(f_{1},\ldots ,f_{N},\mu )\in R^{N+1}$ , where $f_{1},\ldots ,f_{N}$ are the relative abundances of the organisms (with $\sum_{i=1}^{N}f_{i}=1$ ) and μ is the growth rate of the community (15). Each pair (F,μ) defines a polytope $P_{F,\mu }$ of flux distributions satisfying all constrains of the model for the abundance F and growth rate μ . We say that the pair (F,μ) is *feasible* if given F and μ , at least one flux distribution satisfies all constraints of the community model, that is, $P_{F,\mu }\neq \emptyset$ . We define the abundance-growth space as the set of all feasible abundance-growth pairs. We use the term “space” to remark that each point (F,μ) is a representation of the set of $P_{F,\mu }$ of feasible fluxes. It is important to note that, since the constraints related to the flux of biomass of each organism are not linear when both F and μ are variables, then the set of feasible abundance-growth points is not necessarily convex, as shown in the synthetic *E. coli* community example. However, for fixed values of the vector F or μ they become linear, and so, the convexity is assured. This means that convexity is assured in horizontal and vertical sections of the abundance-growth space.

*Defining a grid of feasible abundance-growth points*. A set ℱ , corresponding to a discretization of the abundance-growth space, is defined as follows. Specifically, for a given resolution parameter ℓ , we define a discretization of the possible relative abundances values by considering the set $\ddot{\bar{\left[0,1\right]}}$ corresponding to ℓ equidistant values in the interval [0,1] . Thus, the set A of relative abundance points $\left(f_{1},\ldots ,f_{N}\right)$ that we consider is defined by:


$$A=\left\{\left(f_{1},\ldots ,f_{N}\right)\in {\ddot{\bar{\left[0,1\right]}}}^{N}|\sum_{i=1}^{N}f_{i}=1\right\}.$$

A second discretization is done for the community growth rate μ by defining the set $\ddot{\bar{\left[0,MAX_{biomass}\right]}}$ corresponding to ℓ equidistant values in the interval $\left[0,MAX_{biomass}\right]$ , where $MAX_{biomass}$ is the maximum biomass computed in the model for all abundance points in A . The set of abundance-growth points considered in the analysis is defined by:


$$A\times G=\{(f_{1},\ldots ,f_{N},\mu )\in {\ddot{\bar{\left[0,1\right]}}}^{N}\times \ddot{\bar{\left[0,{MAX}_{biomass}\right]}}\;|\sum_{i=1}^{N}f_{i}=1\}.$$

Finally, the set of points that define the grid ℱ are those that are feasible. That is, the points $\left(f_{1},\ldots ,f_{N},\mu \right)$ in A×G such that the community can reach a growth μ for the given relative abundances $\left(f_{1},\ldots ,f_{N}\right)$ . Note that, since $\sum_{i=1}^{N}f_{i}=1$ , then an abundance of one organism can be omitted in an abundance-growth point, which is especially useful in the case when N=2 , since this implies that abundance-growth points $\left(f_{1},\mu \right)$ can be depicted in a two-dimensional plot.

*Computing qualitative vectors for the abundance-growth points*. Each point (F,μ) of the abundance-growth space is a representation of all the flux distributions in its associated polytope $P_{F,\mu }$ . Given a point (F,μ)∈ℱ in the abundance-growth space, the range of feasible fluxes of a reaction r is exactly the interval $\left[{min}_{F,\mu }(r),{max}_{F,\mu }(r)\right]$ , where $min(r)=min\{v_{r}\;|\text{ for all }v\in P_{F,\mu }\}$ , $max(r)=max\{v_{r}\;|\text{ for all }v\in P_{F,\mu }\}$ . Both amounts are easily determined in the community model by running flux variability analysis (FVA) (31) fixing F and μ in the constraint-based model. Thus, the interval $\left[{min}_{F,\mu }(r),{max}_{F,\mu }(r)\right]$ is computed for all reactions r in each point (F,μ) of the grid ℱ . Computation of these amounts can be done in parallel or by efficiently implementing consecutive LP formulations with the same feasible space (58). The range $\left[{min}_{F,\mu }(r),{max}_{F,\mu }(r)\right]$ is used to give a categorical value to each reaction r and abundance-growth point (F,μ) (Table 1). These qualitative values are encapsulated in the vector $X\,\left(F,\mu \right)\in C^{\left|R\right|}$ of dimension equal to the number of analyzed reactions in the community model, where C is the set of categories. In the applications of this method, a subset $R^{\prime }\subset R$ of selected reactions will be considered, and thus we will focus our analysis on the restriction of X⁢(F,μ) to the coordinates associated with $R^{\prime }$ , say $X^{\prime }\,\left(F,\mu \right)\in C^{\left|R^{\prime }\right|}$ .

*Partition and cluster-partition of the abundance-growth space*. As stated previously, the vectors $X^{\prime }\,\left(F,\mu \right)\in C^{\left|R^{\prime }\right|}$ are qualitative descriptions of the set of reactions on each point (F,μ) of the grid ℱ . We define two ways of partitioning this grid. First, using the categorical vectors $X^{\prime }\,\left(F,\mu \right)$ , a partition of the grid can be defined by stating that two feasible abundance-growth points in the grid $\left(F_{1},\mu_{1}\right)$ and $\left(F_{2},\mu_{2}\right)$ are equivalent if $X^{\prime }\,\left(F_{1},\mu_{1}\right)=X^{\prime }\,\left(F_{2},\mu_{2}\right)$ . Thus, each set of the partition can be considered a zone where selected reactions in the model have identical categorical descriptions. It is called the partition of the abundance-growth space defined by the selected reactions in $R^{\prime }$ .

Depending on the size of the set of selected reactions $R^{\prime }$ , the number of zones in this partition can be huge. To address this issue, we propose to use a coarser partition based on the degree of similarity of the categorical vectors. More precisely, we perform a classification of the vectors $X^{\prime }\,\left(F,\mu \right)$ for each (F,μ)∈ℱ and $r\in R^{\prime }$ using a hierarchical clustering algorithm with a Jaccard distance, where the number of clusters ( k ) is defined *a priori*. In other words, we can identify zones of the grid where reactions have a similar categorical behavior according to the clustering method used. We call this partition the cluster-partition of the abundance-growth space (defined by the selected reactions in $R^{\prime }$ ). Alternatively, this method can be implemented using other clustering methods.

Additionally, we want to summarize the qualitative state for each reaction on a given zone defined by a cluster partition. To achieve this, for each reaction, given a cluster, we assign a qualitative value to each reaction if such a qualitative state is present in over 80 % of the points of that cluster.

*Determination of the number of clusters for analysis*. Determination of the number of clusters to be used is performed as follows: for a given number k of clusters, each categorical vector $X^{\prime }\,\left(F,\mu \right)$ is compared to its assigned cluster-partition descriptor. For each reaction, a score value between 0 and 1 represents the fraction of points consistent with the cluster-partition descriptor. This analysis is performed with different values of k clusters to retrieve the minimum number of clusters where all analyzed reactions are represented correctly in at least 80 % of points of the grid.

*Quantitative flux coupling analysis*. Given an abundance-growth point, the space of feasible fluxes for two given reactions *r*_1_ and *r*_2_ is computed by defining a homogeneous grid of 50 values between the minimum and maximum value of the flux of *r*_1_ computed by FVA. Then, for each flux value of *r*_1_ in this grid, an FVA is performed for reaction *r*_2_ to compute the minimum and maximum flux values of *r*_2_. The interval defined by these values is the feasible fluxes of *r*_2_ for the given flux value of *r*_1_. Normalization by *f*_*i*_ is performed to retrieve flux values in units of (mmol/g DW_*i*_/h) for each analyzed reaction.

### Analysis of a synthetic *E. coli* community

A community defined by two strains of *Escherichia coli* auxotrophic for leucine and lysine was selected from reference 37. The *E. coli* genome-scale model iAF1260 (59) was modified by constraining reactions associated to gene knock-outs made in reference 37: diaminopimelate decarboxylase (DAPDC) for the lysine auxotrophic strain (eco_K) and 2-isopropylmalate synthase (IPPS) for the leucine auxotrophic strain (eco_L). Glucose availability was set up to be 10 (mmol/g DW_ ${}_{o\,r\,g}$ _/h), amino acid production was set up to be 0.2 (mmol/g DW_ ${}_{o\,r\,g}$ _/h) for both leucine and lysine, and consumption for both amino acids for both strains was constrained according to reported values of production and consumption stated in reference (37). A grid of ℓ=50 points is generated according to what was described previously, obtaining that from the 2,500 potential points of the grid, only 973 are feasible given how consumption and production constraints set up for this model are affected by community composition (File S1, https://github.com/mathomics/ecosystem). If amino acid availability is simulated, an external supplementation of 0.01 (mmol/g DW_ ${}_{org}$ _/h) for both leucine and lysine is added by modifying the lower bound of exchanges for these amino acids.

### Analysis of bioleaching community

*Reconstruction of genome-scale models for bioleaching organisms*. Two genome-scale models were reconstructed for *Acidithiobacillus ferrooxidans* Wenelen (iML510) and *Sulfobacillus thermosulfidooxidans* Cutipay (ISM517). Genome sequences for *A. ferrooxidans* Wenelen and *S. thermosulfidooxidans* Cutipay were retrieved from references 60 – 62. The AuReMe workflow (63, 64) was used for the independent reconstruction of each metabolic network. For *S. thermosulfidooxidans* Cutipay, a model previously reconstructed in reference 63 for this bacteria was used as a starting point for manual curation.

For *A. ferrooxidans*, a draft reconstruction was first obtained using reference metabolic models from other species and gene orthology (65). For *A. ferrooxidans* Wenelen, the model iMC507 for *A. ferrooxidans* ATCC23270 (66) was used as a reference (67).

Drafts for both *A. ferrooxidans* Wenelen and *S. thermosulfidooxidans* Cutipay were manually curated. Particular attention was given to iron and sulfur oxidation metabolism (68, 69). Pathways from these subsystems were completed after including reactions from the literature (File S3, https://github.com/mathomics/ecosystem) (68 – 71). Since no biomass composition information was available for either of the bacteria studied, adaptations of biomass functions from models iMC507 and iHN637 were used for Wenelen and Cutipay, respectively. Both models were checked using Memote to ensure their quality before publication (72, 73).

Final metabolic models for *A. ferrooxidans* Wenelen (iML510) and *S. thermosulfidooxidans* Cutipay (iSM517) are available at the following address: https://github.com/mathomics/ecosystem and in File S3. The obtained models display the associations between 495 genes for *S. thermosulfidooxidans* and a metabolic network comprising 985 metabolites and 1,056 reactions. On the other hand, the genome-scale model for *A. ferrooxidans* includes 506 genes associated with 612 reactions and 579 metabolites.

Single genome-scale models for *A. ferrooxidans* Wenelen and *S. thermosulfidooxidans* Cutipay were adjusted to exhibit realistic growth rates in simulations with FBA under the presence of iron [Fe(II)] or sulfur (thiosulfate) as energy sources. For this purpose, lower bounds associated with exchanging reactions for Fe(II) and thiosulfate were modified to retrieve growth rates reported in the literature, particularly a maximum growth rate of 0.15 per hour (49, 66, 74).

*Metabolic model of the bioleaching community*. The individual models previously reconstructed were merged to represent a bioleaching community model in which there is competition for carbon dioxide consumption as well as two external resources: iron [Fe(II)] and sulfur (thiosulfate). Single-entry fluxes for these compounds are supplied in a pool compartment to which both bacteria have access and represent their growth environment. The total availability of these resources was modeled by setting the lower bound of the exchange reactions of the community (EX_fe2_e, EX_tsul_e) depending on different analyzed scenarios described in the next section. Iron and sulfur direct assimilations as part of biomass composition for both bacteria were represented by the metabolites fe2aa (iron assimilation) and so4aa (sulfate assimilation), respectively, to differentiate them from iron and sulfur consumption for energy production. These metabolites are available in non-growth-limiting entry fluxes for both members of the community. Other nutrients required for growth were modeled as equally available for both bacteria.

The capacity of Cutipay to use the degraded organic matter of the community as an additional energy source is modeled as an incorporation of the following pseudo-reactions representing the degradation of community biomass and its transformation into carbon equivalent units of glucose:


$$Biomass_{community}\to \alpha Biomass_{degraded}+(1-\alpha )Biomass_{net}$$

$${Biomass}_{degraded}\to 6.169glc^{pool}$$

where parameter α represents that the death rate of the organisms is equivalent to a fraction α of the community growth rate. The choice of glucose as a unique representation of organic matter is an arbitrary simplification in the absence of more information about how the organisms are degraded. This was done because glucose can be easily assimilated by the metabolic network (File S3, https://github.com/mathomics/ecosystem), as well as that it can be incorporated to synthesize nucleotides, amino acids, as well as other simpler organic acids found in bioleaching environments, such as acetate.

The set of 1,717 reactions of this community model comprises 1,668 reactions from the original single models and 49 reactions that appear in the construction of the community model. The set of 1,611 metabolites of the community model comprises 1,564 metabolites from single models and 47 metabolites from the community construction.

*Definition of parameters and scenarios for simulations of bioleaching communities*. Different external and internal conditions are explored to determine how these scenarios change the distribution of qualitative states in the abundance-growth space. External conditions are represented by available resources, both organic and inorganic, for the community. Meanwhile, internal conditions correspond to alternative requirements for the obtained flux distributions, mainly being energetically efficient.

Modeling the availability of organic matter is achieved by setting the parameter α . The availability of inorganic sources is modeled, defining the available fluxes of Fe(II) and thiosulfate in the community. It is achieved by adjusting the lower bounds for the community exchange reactions of both sources.

Thus, we define the values ${\text{Max}}_{\text{Fe(II)}}=$ 150 (mmol/g DW_ ${}_{c\,o\,m}$ _/h) and ${\text{Max}}_{\text{tsul}}=$ 10 (mmol/g DW_ ${}_{c\,o\,m}$ _/h) as reference values, which correspond to the uptakes of Fe(II) and thiosulfate that produce the maximum level of biomass on both organisms according to their single models.

Any convex combination of these amounts generates an equivalent amount of inorganic sources. Formally, a relative combination of λ Fe(II) and 1-λ thiosulfate means that there is a flux of $\lambda \,{\text{Max}}_{\text{Fe(II)}}$ of Fe(II) and a flux of $\left(1-\lambda \right)\,{\text{Max}}_{\text{tsul}}$ of thiosulfate available to the community. With these definitions, the following scenarios are defined:

1. A *reference case* is defined as having a low organic availability ( α=0.2 ), and a relative combination of 0.5 Fe(II) and 0.5 thiosulfate ( λ=0.5 ).
2. Analysis of the effects of organic matter is performed by changing α in the reference case scenario ( λ=0.5 ). The analyzed values of α are 0, 0.2, 0.4, 0.6, and 0.8.
3. Analysis of the effect of substrate composition is performed by variations in the value of λ in the reference case ( α=0.2 ). The analyzed values of λ are 0.25, 0.4, 0.5, 0.6, and 0.75.
4. Analysis of alternative objective functions is performed by analyzing the reference case ( α=0.2 , λ=0.5 ) when additional functions are required. Specifically, energetic efficiency is represented as an additional constraint which states that the sum of fluxes of all reactions is less than a factor of the minimum sum of fluxes. The analyzed values for this factor are 1.1, 1.2, and 1.5.

## Data Availability

This method was implemented in Python using COBRApy for constructing community models, FBA, and FVA simulations (75), and it is available at Github.

## SUPPLEMENTAL MATERIAL

Fig. S1 msystems.00492-23-s0001.pdf Effect of modifying production capacity and supplementation of leucine and lysine in the abundance-growth space of the *E. coli* synthetic community.

Fig. S2 msystems.00492-23-s0002.pdf Partition of the abundance-growth space determined by exchange of leucine and lysine in a synthetic *E. coli* community.

Fig. S3 msystems.00492-23-s0003.pdf Determination of the number of clusters for a synthetic *E. coli* community.

Fig. S4 msystems.00492-23-s0004.pdf Partition of the abundance-growth space determined by key reactions in a synthetic *E. coli* community.

Fig. S5 msystems.00492-23-s0005.pdf Effect of modifying leucine and lysine supplementation in leucine production and export by eco_K.

Fig. S6 msystems.00492-23-s0006.pdf Quantitative flux coupling analysis for exchanges of lysine and leucine in the synthetic *E. coli* supplemented with these amino acids.

Fig. S7 msystems.00492-23-s0007.pdf Effect of the variation of the number of clusters in the bioleaching community.

Fig. S8 msystems.00492-23-s0008.pdf Partition of the abundance-growth space determined by 11 selected key bioleaching reactions describes relevant shifts in the bioleaching community

Fig. S9 msystems.00492-23-s0009.pdf Quantitative flux coupling analysis for iron(II) competition in the bioleaching community.

Fig. S10 msystems.00492-23-s0010.pdf Quantitative flux coupling analysis for the distribution of resources by *S. thermosulfidooxidans* in the bioleaching community.

ASM does not own the copyrights to Supplemental Material that may be linked to, or accessed through, an article. The authors have granted ASM a non-exclusive, world-wide license to publish the Supplemental Material files. Please contact the corresponding author directly for reuse.
