# Supplementary material for: Unveiling abundance-dependent metabolic phenotypes of microbial communities
Source: mSystems. 2023 Sep 5;8(5):e00492-23. doi: 10.1128/msystems.00492-23 (PMC10654064; doi:10.1128/msystems.00492-23)
Supplement: Fig. S1 — Effect of modifying production capacity and supplementation of leucine and lysine in the abundance-growth space of the E. coli synthetic community. [file msystems.00492-23-s0001.pdf]

(a)

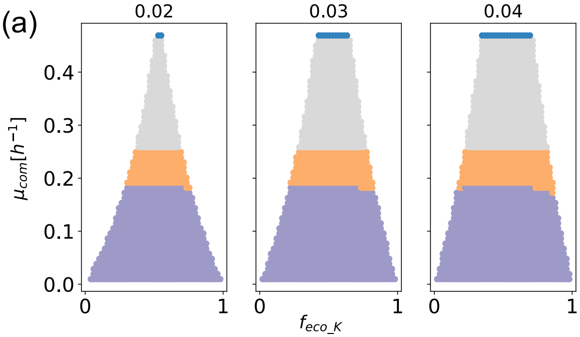

EX_co2_e	++	-+	-+	-+
EX_o2_e	--	--	-0	--
EX_h2o_e	++	-+	-+	++
EX_lys__L_e	0+	0+	0+	0+
EX_leu__L_e	0+	0+	0+	0+

(b)

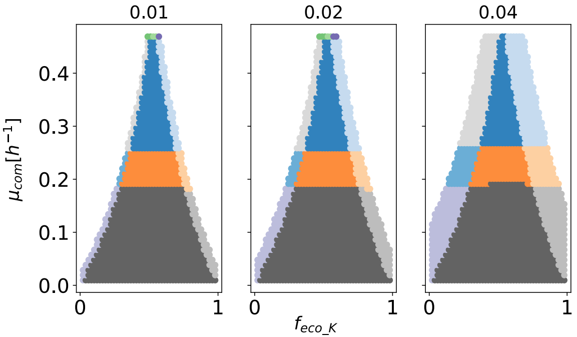

EX_co2_e	-+	-+	-+	-+	-+	++	++	++	-+	-+	-+	-+
EX_o2_e	--	--	--	--	--	--	--	--	-0	-0	-0	--
EX_h2o_e	++	-+	++	-+	-+	++	++	++	-+	-+	-+	++
EX_lys__L_e	-+	-+	--	-+	--	-+	-+	--	-+	-+	--	-+
EX_leu__L_e	-+	--	-+	-+	-+	--	-+	-+	--	-+	-+	--

Figure S1. Effect of modifying production capacity and supplementation of leucine and lysine in the abundance-growth space of the *E. coli* synthetic community. (a) For three different maximum production capacity of leucine and lysine in both bacteria (0.02, 0.03 and 0.04 [mmol $gDWcom^{-1}$ h^{-1}]) the partitions associated to exchange reactions are depicted together with the table of their qualitative states. (b) For three different supplementation of leucine and lysine to the community (0.01, 0.02 and 0.04 [mmol $gDWcom^{-1}$ h^{-1}]) the partitions associated to exchange reactions are depicted together with the table of their qualitative states.
