# Supplementary material for: Unveiling abundance-dependent metabolic phenotypes of microbial communities
Source: mSystems. 2023 Sep 5;8(5):e00492-23. doi: 10.1128/msystems.00492-23 (PMC10654064; doi:10.1128/msystems.00492-23)
Supplement: Fig. S2 — Partition of the abundance-growth space determined by exchange of leucine and lysine in a synthetic E. coli community. [file msystems.00492-23-s0002.pdf]

(a)

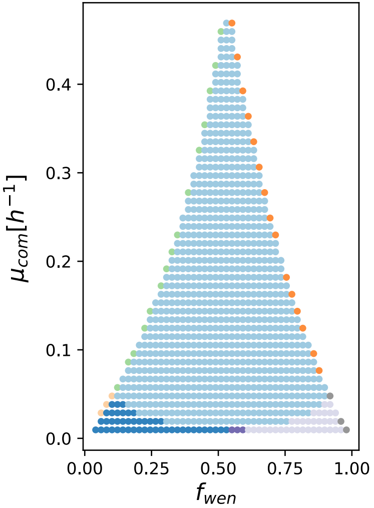

(b)

**eco_K_EX_leu__L_e**	+	++	0+	0+	0+	0+	++	+
**eco_L_EX_lys__L_e**	0+	0+	0+	++	++	++	++	+

Figure S2. Partition of the abundance-growth space determined by exchange of leucine and lysine in a synthetic *E. coli* community. (a) A partition of the abundance-growth space for exchange reactions of lysine supplementation by eco_L (EX_eco_L_lys__L_e) and leucine supplementation by eco_K (EX_eco_K_leu__L_e). (b) Table showing qualitative states of both reactions in the abundance-growth space.
