# Supplementary material for: Unveiling abundance-dependent metabolic phenotypes of microbial communities
Source: mSystems. 2023 Sep 5;8(5):e00492-23. doi: 10.1128/msystems.00492-23 (PMC10654064; doi:10.1128/msystems.00492-23)
Supplement: Fig. S3 — Determination of the number of clusters for a synthetic E. coli community. [file msystems.00492-23-s0003.pdf]

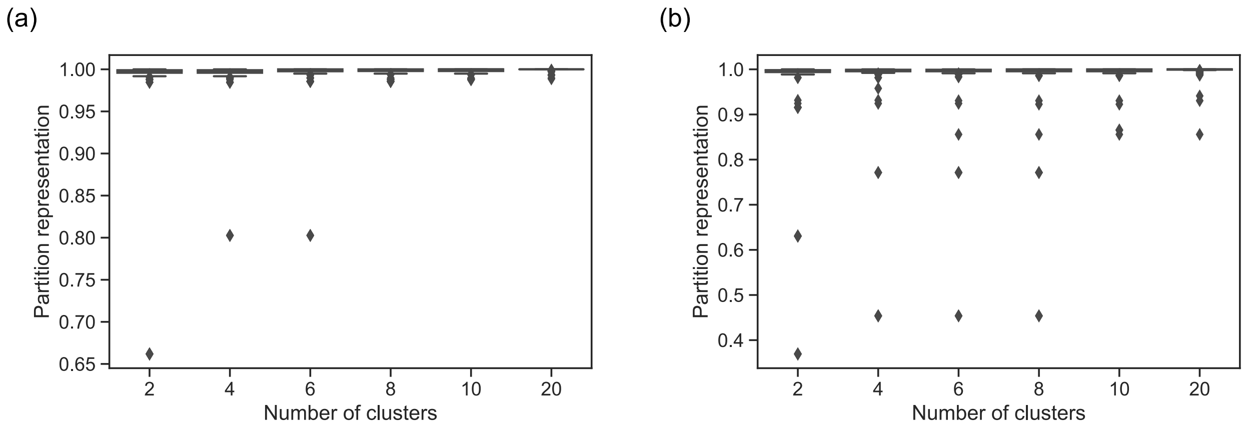

Figure S3. Determination of the number of clusters for a synthetic *E. coli* community. In two scenarios, we depict the percentage of grid points where their qualitative vector values coincide with the consensus ones versus the number of clusters. The choice of the number of clusters to be used is the one that accurately represents over 80% of the points of each reaction on the grid. (a) In the scenario with no external lysine or leucine supplementation we choose 8 clusters. (b) In the scenario where both leucine and lysine are supplemented we choose 10 clusters.
