# Supplementary material for: Unveiling abundance-dependent metabolic phenotypes of microbial communities
Source: mSystems. 2023 Sep 5;8(5):e00492-23. doi: 10.1128/msystems.00492-23 (PMC10654064; doi:10.1128/msystems.00492-23)
Supplement: Fig. S4 — Partition of the abundance-growth space determined by key reactions in a synthetic E. coli community. [file msystems.00492-23-s0004.pdf]

(a)

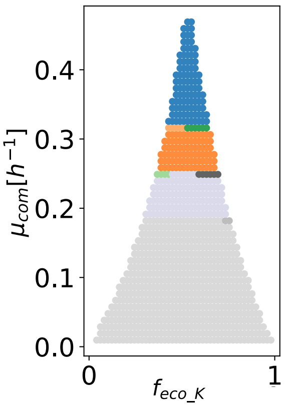

(b)

EX_h2o_e	-+	-+	-+	-+	-+	++	++	++	++
EX_o2_e	-0	--	--	--	--	--	--	--	--
eco_K_ATPS4rpp	-+	-+	-+	-+	-+	-+	-+	-+	++
eco_K_EX_h2o_e	-+	-+	-+	++	-+	++	++	++	++
eco_K_EX_o2_e	-0	-0	--	--	--	--	--	--	--
eco_K_RPI	-+	-+	-+	-+	-+	-+	--	--	--
eco_L_ATPS4rpp	-+	-+	-+	-+	-+	-+	-+	++	++
eco_L_EX_h2o_e	-+	-+	-+	-+	++	++	++	++	++
eco_L_EX_o2_e	-0	--	--	--	--	--	--	--	--
eco_L_RPI	-+	-+	-+	-+	-+	-+	--	--	--

Figure S4. Partition of the abundance-growth space determined by key reactions in a synthetic *E. coli* community. (a) Partition of the abundance-growth space is computed for 10 reactions determined in the analysis of the cluster-partition for a *E. coli* synthetic community. (b) Table with the qualitative state of the key reactions used to compute the partition. Reactions with EX prefix denote exchange reactions. Partitions are organized according to their location in the abundance-growth space from low to high growth rate and from f_{eco_K} 0 to 1. EX: exchange reactions, h2o: water, o2: oxygen, lys: lysine, leu: leucine, ATPS4rpp: ATP synthase, RPI: Ribose-5-phosphate isomerase
