# Supplementary material for: Unveiling abundance-dependent metabolic phenotypes of microbial communities
Source: mSystems. 2023 Sep 5;8(5):e00492-23. doi: 10.1128/msystems.00492-23 (PMC10654064; doi:10.1128/msystems.00492-23)
Supplement: Fig. S5 — Effect of modifying leucine and lysine supplementation in leucine production and export by eco_K. [file msystems.00492-23-s0005.pdf]

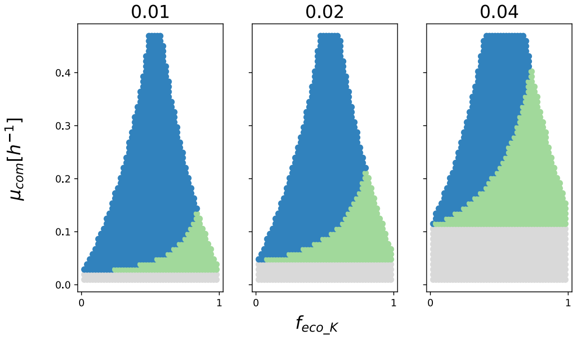

eco_K_IPPS	++	++	0+
eco_K_EX_leu__L_e	++	-+	-+

Figure S5. Effect of modifying leucine and lysine supplementation in leucine production and export by eco_K. For three different supplementation of leucine and lysine to the *E. coli* community (0.01, 0.02 and 0.04 [mmol gDWcom^{-1} h^{-1}]) the partitions associated to leucine production and export by eco_K are depicted together with the table of their qualitative states.
