# Supplementary material for: Unveiling abundance-dependent metabolic phenotypes of microbial communities
Source: mSystems. 2023 Sep 5;8(5):e00492-23. doi: 10.1128/msystems.00492-23 (PMC10654064; doi:10.1128/msystems.00492-23)
Supplement: Fig. S6 — Quantitative flux coupling analysis for exchanges of lysine and leucine in the synthetic E. coli supplemented with these amino acids. [file msystems.00492-23-s0006.pdf]

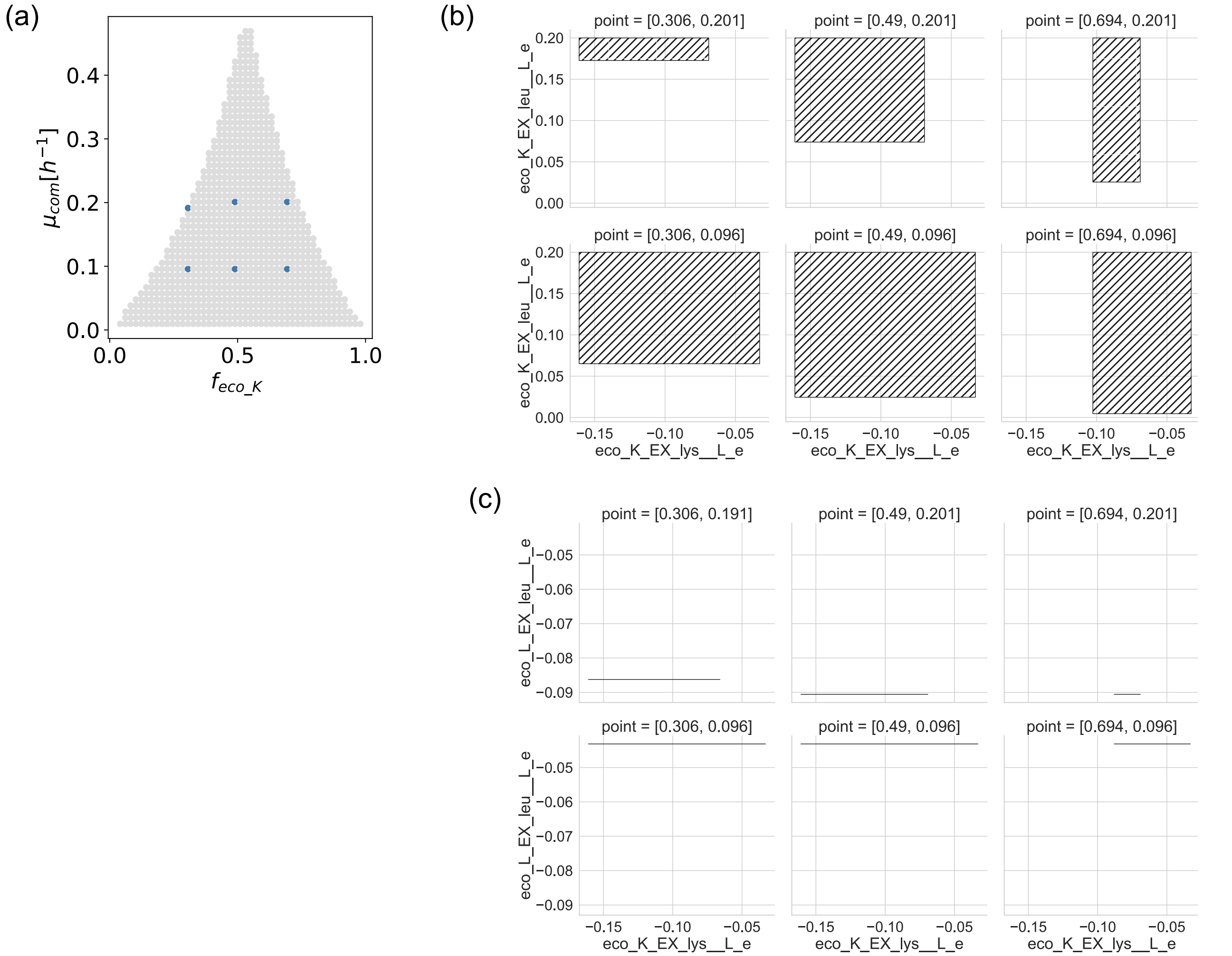


**Figure S6. Quantitative flux coupling analysis for exchanges of lysine and leucine in the synthetic *E. coli* supplemented with these amino acids.** (a) Selected points in the abundance-growth space. (b) Feasible fluxes for consumption of lysine and production of leucine by eco_K at the different points marked in (a). (c) Feasible fluxes for consumption of lysine by eco_K and consumption of leucine by eco_L. Fluxes are in units of [mmol/ $gDW_{org}$ $h^{-1}$].
