# Supplementary material for: Unveiling abundance-dependent metabolic phenotypes of microbial communities
Source: mSystems. 2023 Sep 5;8(5):e00492-23. doi: 10.1128/msystems.00492-23 (PMC10654064; doi:10.1128/msystems.00492-23)
Supplement: Fig. S7 — Effect of the variation of the number of clusters in the bioleaching community. [file msystems.00492-23-s0007.pdf]

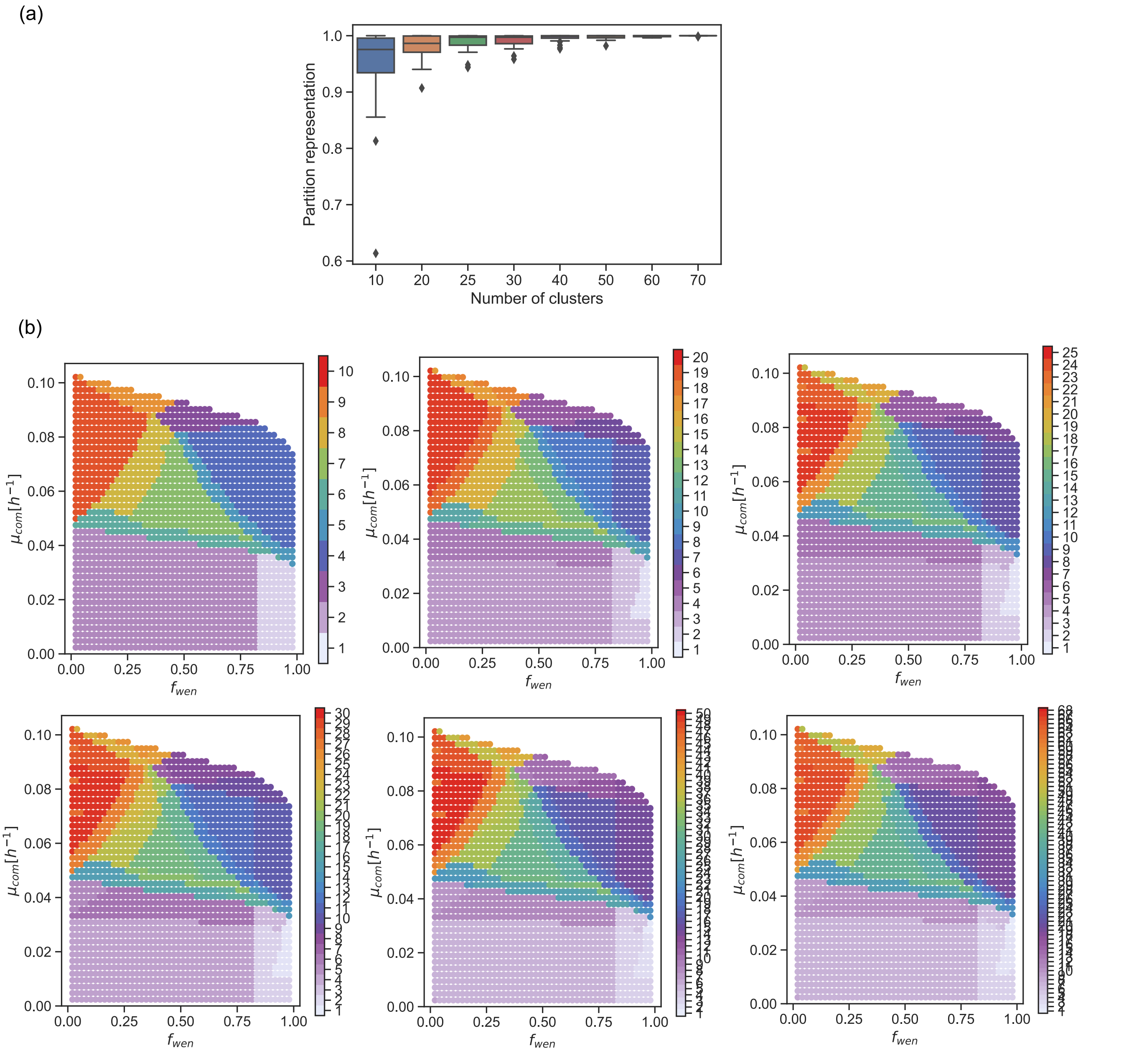

Figure S7. Effect of the variation of the number of clusters in the bioleaching community. (a) Partition representation improves by increasing the number of clusters. When the number of clusters is 20 all reactions are accurately represented in at least 90% of the points of the grid. (b) Zones of the abundance-growth space obtained when moving the number of clusters from 10 to 69.
