# Supplementary material for: Unveiling abundance-dependent metabolic phenotypes of microbial communities
Source: mSystems. 2023 Sep 5;8(5):e00492-23. doi: 10.1128/msystems.00492-23 (PMC10654064; doi:10.1128/msystems.00492-23)
Supplement: Fig. S8 — Partition of the abundance-growth space determined by 11 selected key bioleaching reactions describes relevant shifts in the bioleaching community [file msystems.00492-23-s0008.pdf]

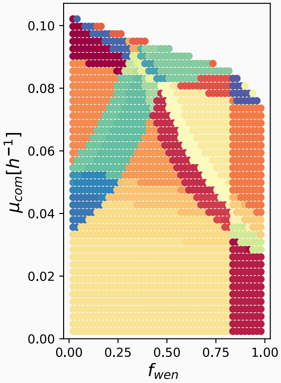

	High plasticity							Proton plasticity					
EX_fe2_e	-0	-0	-0	-0	-0	-0	-0	--	--	--	--	--	--
EX_tsul_e	-0	-0	--	--	--	--	--	--	--	--	--	--	--
EX_h_e	-+	-+	-+	-+	-+	-+	-+	-+	-+	-+	-+	-+	-+
cut_EX_fe2_e	-0	-0	-0	-0	-0	-0	-0	--	-0	-0	-0	-0	-0
cut_EX_tsul_e	-0	-0	--	-0	-0	-0	-0	--	--	-0	-0	-0	-0
cut_EX_h_e	-+	-+	-+	-+	-+	-+	-+	-+	-+	-+	-+	-+	-+
cut_EX_glc_e	-0	-0	-0	-0	-0	-0	-0	-0	-0	-0	-0	-0	-0
cut_EX_co2_e	--	-+	--	--	--	-+	-+	--	--	--	--	-+	-+
wen_EX_fe2_e	-0	-0	-0	-0	-0	-0	-0	-0	-0	-0	--	--	-0
wen_EX_tsul_e	-0	-0	-0	-0	--	--	-0	-0	-0	--	--	--	--
wen_EX_h_e	-+	-+	-+	-+	-+	-+	-+	-+	-+	-+	-+	-+	-+

	Wenelen plasticity				CW-P		Cutipay plasticity				
EX_fe2_e	--	--	--	--	--	--	--	--	--	--	--
EX_tsul_e	--	--	--	--	--	--	--	--	--	--	--
EX_h_e	--	--	--	--	--	-+	--	--	--	--	--
cut_EX_fe2_e	--	--	-0	-0	-0	-0	-0	-0	-0	-0	-0
cut_EX_tsul_e	--	--	--	--	-0	-0	-0	-0	-0	-0	-0
cut_EX_h_e	--	-+	-+	-+	-+	-+	-+	-+	-+	-+	-+
cut_EX_glc_e	-0	-0	-0	-0	-0	-0	-0	-0	-0	-0	-0
cut_EX_co2_e	--	--	--	--	--	--	--	--	--	-+	-+
wen_EX_fe2_e	-0	-0	-0	-0	-0	-0	-0	--	--	--	--
wen_EX_tsul_e	-0	-0	-0	--	-0	-0	--	--	--	--	--
wen_EX_h_e	-+	-+	-+	-+	-+	-+	-+	--	-+	-+	--

	Low plasticity															
EX_fe2_e	--	--	--	--	--	--	--	--	--	--	--	--	--	--	--	--
EX_tsul_e	--	--	--	--	--	--	--	--	--	--	--	--	--	--	--	--
EX_h_e	--	--	--	--	--	--	--	--	--	--	--	--	--	--	--	--
cut_EX_fe2_e	--	--	-0	--	-0	--	-0	--	-0	-0	-0	--	--	--	-0	-0
cut_EX_tsul_e	--	--	--	--	--	--	--	--	--	-0	-0	--	--	--	-0	--
cut_EX_h_e	--	-+	-+	-+	-+	-+	-+	-+	-+	-+	-+	--	--	--	-+	++
cut_EX_glc_e	--	--	--	--	--	--	--	--	--	--	--	--	--	--	--	--
cut_EX_co2_e	--	--	--	--	--	--	--	--	--	--	-+	--	--	--	++	--
wen_EX_fe2_e	-0	-0	-0	-0	-0	--	--	--	--	--	--	-0	--	--	--	--
wen_EX_tsul_e	-0	-0	-0	--	--	--	--	--	--	--	--	--	--	--	--	--
wen_EX_h_e	-+	-+	-+	-+	-+	-+	-+	--	--	--	--	-+	-+	--	--	--

Figure S8. Partition of the abundance-growth space determined by 11 selected key bioleaching reactions describes relevant shifts in the bioleaching community. The partition of the abundance-growth space determined from reactions for oxidation of iron and sulfur and carbon metabolism shows that critical exchange reactions associated with energy resources and carbon metabolism can fully characterize the metabolic shifts occurring in the bioleaching community, yielding 40 areas with defined qualitative status. Reactions with EX prefix denote exchange reactions, cut: *S. thermosulfidooxidans* Cutipay, wen: *A. ferrooxidans* Wenelen, co2: carbon dioxide, fe2: Fe(II), glc: glucose, h: hydrogen, tsul: thiosulfate, CW-P: Cutipay Wenelen plasticity. Partitions are organized according to their location in the abundance-growth space from lower to high growth rate and from f_{wen} 0 to 1.
