# Supplementary figures and images for: Unveiling abundance-dependent metabolic phenotypes of microbial communities
Source: mSystems. 2023 Sep 5;8(5):e00492-23. doi: 10.1128/msystems.00492-23 (PMC10654064; doi:10.1128/msystems.00492-23)

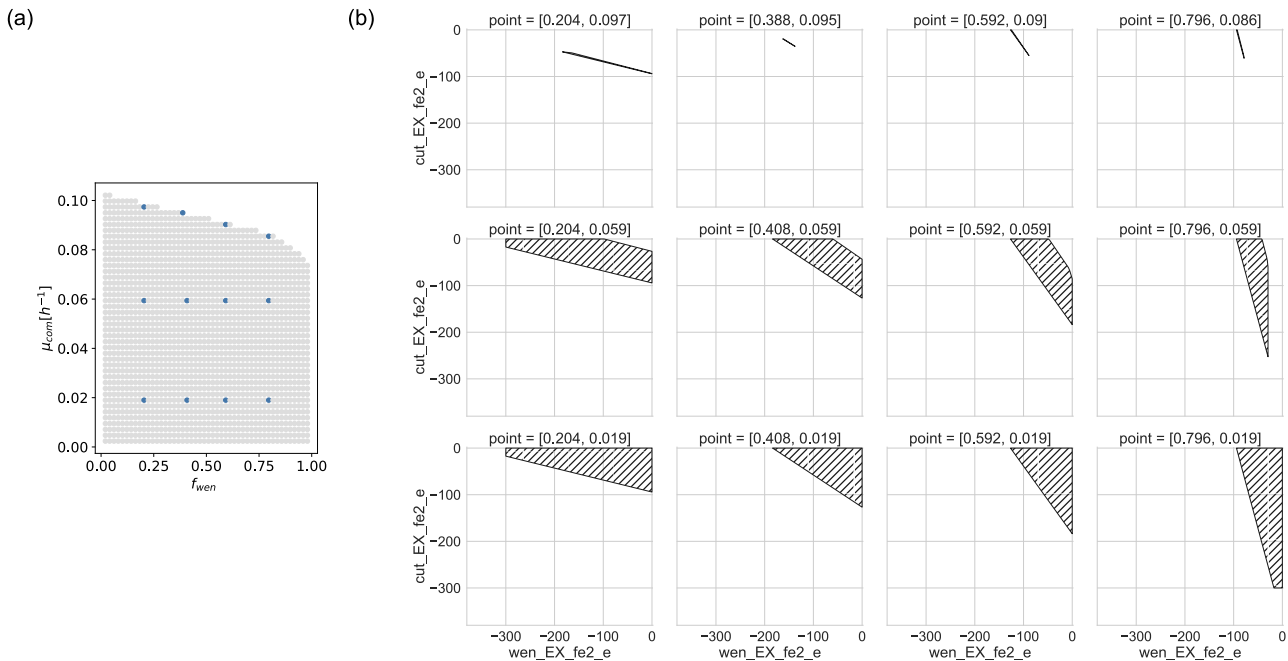

Supplement: Fig. S9 — Quantitative flux coupling analysis for iron(II) competition in the bioleaching community. [file msystems.00492-23-s0009.pdf]
