# Supplementary material for: Unveiling abundance-dependent metabolic phenotypes of microbial communities
Source: mSystems. 2023 Sep 5;8(5):e00492-23. doi: 10.1128/msystems.00492-23 (PMC10654064; doi:10.1128/msystems.00492-23)
Supplement: Fig. S10 — Quantitative flux coupling analysis for the distribution of resources by S. thermosulfidooxidans in the bioleaching community. [file msystems.00492-23-s0010.pdf]

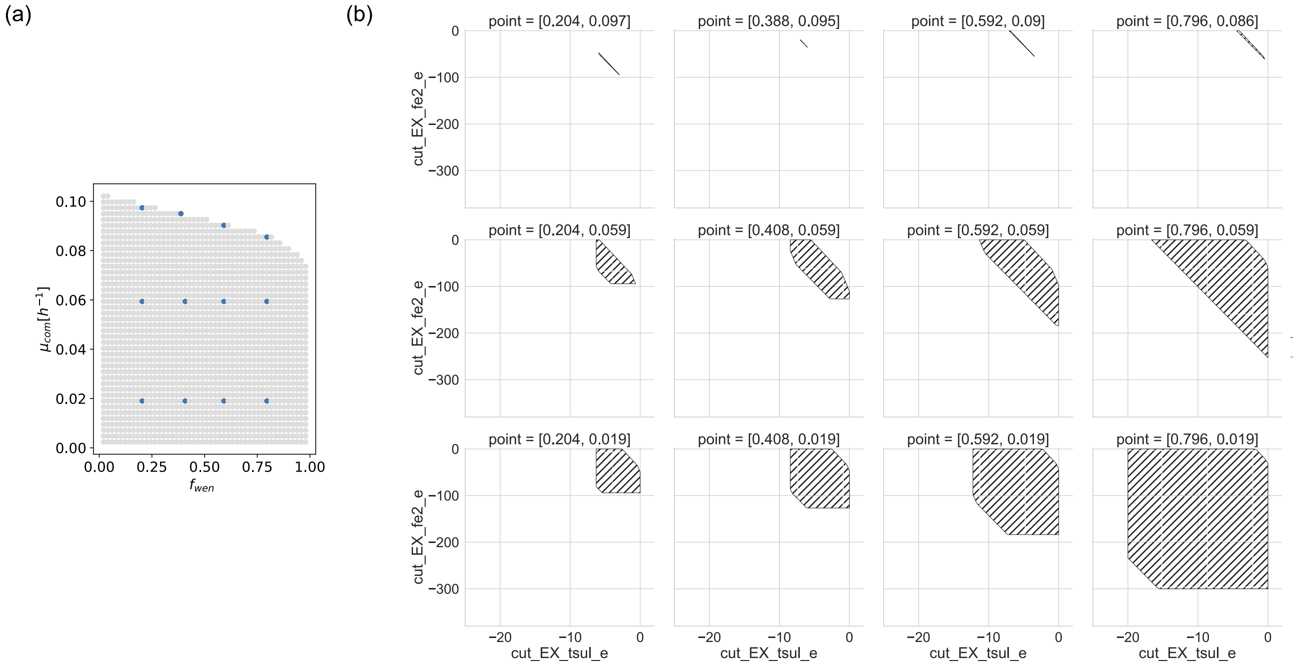

Figure S10. Quantitative flux coupling analysis for the distribution of resources by *S. thermosulfidooxidans* in the bioleaching community. (a) Selected points in the abundance-growth space. (b) Feasible fluxes of Fe(II) and thiosulfate exchanges (EX_fe2_e and EX_tsul_e) for *S. thermosulfidooxidans* at the different points marked in (a). Fluxes are in units of [mmol/ gDW_{cut} h^{-1}].
